# Deep-sea mussels from a hybrid zone on the Mid-Atlantic Ridge host genetically indistinguishable symbionts

**DOI:** 10.1038/s41396-021-00927-9

**Published:** 2021-05-10

**Authors:** Merle Ücker, Rebecca Ansorge, Yui Sato, Lizbeth Sayavedra, Corinna Breusing, Nicole Dubilier

**Affiliations:** 1 Max Planck Institute for Marine Microbiology, Bremen, Germany; 2 MARUM—Center for Marine Environmental Sciences of the University of Bremen, Bremen, Germany; 3 Quadram Institute Bioscience, Norwich, Norfolk UK; 4 University of Rhode Island, Graduate School of Oceanography, Narragansett, RI USA

**Keywords:** Metagenomics, Population genetics, Symbiosis, Microbiome

## Abstract

The composition and diversity of animal microbiomes is shaped by a variety of factors, many of them interacting, such as host traits, the environment, and biogeography. Hybrid zones, in which the ranges of two host species meet and hybrids are found, provide natural experiments for determining the drivers of microbiome communities, but have not been well studied in marine environments. Here, we analysed the composition of the symbiont community in two deep-sea, *Bathymodiolus* mussel species along their known distribution range at hydrothermal vents on the Mid-Atlantic Ridge, with a focus on the hybrid zone where they interbreed. In-depth metagenomic analyses of the sulphur-oxidising symbionts of 30 mussels from the hybrid zone, at a resolution of single nucleotide polymorphism analyses of ~2500 orthologous genes, revealed that parental and hybrid mussels (F2–F4 generation) have genetically indistinguishable symbionts. While host genetics does not appear to affect symbiont composition in these mussels, redundancy analyses showed that geographic location of the mussels on the Mid-Atlantic Ridge explained most of the symbiont genetic variability compared to the other factors. We hypothesise that geographic structuring of the free-living symbiont population plays a major role in driving the composition of the microbiome in these deep-sea mussels.

## Introduction

The community composition of an animal’s microbiome is the product of multiple interacting factors that include the environment, geography and host genetics [1–5]. To which extent host genetics affects microbiome composition is currently a topic of intense debate, in part as high-throughput sequencing is revealing the genetic makeup of host and symbiont populations in ever higher resolution [6–8]. Animal hybrids are useful for assessing the effects of host genotype on microbiomes [9]. Studies of lab-reared animal hybrids, such as wasps [10], fish [11–13], and mice [14, 15] found that these hosts had different gut microbiota compositions than their parental species, based on sequencing of the microbial 16S rRNA gene. These altered gut microbiomes of hybrids affected the fitness of some hosts, suggesting that microbiomes play an important role in determining species barriers [10]. Studies on lab-reared hosts cannot, however, fully reflect the environmental conditions animals experience in their natural habitat. Hybrid zones, in which parental species interbreed and produce hybrid offspring, are excellent natural experiments for teasing apart the impact of host genotype, environment and geographic distance on microbiome composition. Yet surprisingly few studies have investigated the microbiota of hybrids from the wild, and these have yielded mixed results. For example, in a hybrid zone of the European house mouse, the composition of gut microbiota of hybrids differed from that of their parental species [15]. In contrast, in African baboons, there were no significant differences between hybrids and their parental species, and gut community composition was best explained by the environment [16]. To date, all hybrid microbiome studies, whether on lab-reared animals or those from the wild, have been based on the sequencing of only a few microbial genes, with the vast majority of analyses based on the 16S rRNA gene, or a variable region of this gene. These studies were therefore limited to determining microbial community composition at the genus level or higher, and could not distinguish closely related species or strains.

Almost nothing is known about the microbial communities of hosts from marine hybrid zones, despite the pervasiveness of such zones in many regions of the oceans. Hydrothermal vents on the Mid-Atlantic Ridge (MAR), an underwater mountain range extending from the Arctic to the Southern Ocean, provide an ideal setting for investigating the microbiomes of hosts in natural hybrid zones. Many of the vents on the MAR are dominated by *Bathymodiolus* mussels that live in a nutritional symbiosis with chemosynthetic bacteria. Two mussel species colonise the northern MAR, *Bathymodiolus azoricus*, which is found at vents from 38° N to 36° N, and *Bathymodiolus puteoserpentis*, which inhabits vents further south from 23° N to 13° N. A hybrid zone between these two relatively young host species, with an estimated splitting time of 8.4 Mya [17], occurs at the Broken Spur vent field at 29° N on the MAR, where *B. puteoserpentis* co-occurs with hybrids between *B. azoricus* and *B. puteoserpentis* [18–20]. The sulphur-oxidising (SOX) symbionts of *B. azoricus* and *B. puteoserpentis* belong to a gammaproteobacterial clade within the *Thioglobaceae*, and co-occur in the mussel gills with methane-oxidising symbionts, which belong to a gammaproteobacterial clade within the *Methylomonaceae* [21]. The relative abundance of these two symbionts in these mussels is assumed to not depend on host genetics, but rather the availability of the energy sources these symbionts use in their environment [21, 22].

The symbionts of bathymodiolin mussels are transmitted horizontally from the environment to juvenile mussels, yet each mussel species harbours a highly specific symbiont community [23–25]. This specificity suggests that the genetics of bathymodiolin mussels plays an important role in determining symbiont composition. In this study, we took advantage of the natural hybrid zone of *Bathymodiolus* mussels at the Broken Spur vent field to investigate how host genotype, geographic distance, and the vent environment affect the composition of their SOX symbionts. The recent discovery of a high diversity of SOX symbiont strains in *Bathymodiolus* from the MAR, with as many as 16 strains co-occurring in single *Bathymodiolus* mussels [22, 26, 27], made it critical to resolve genetic differences at the strain level of the SOX symbiont community (strain is defined here as suggested by Van Rossum et al. [28], as subordinate to subspecies, in our study >99% average nucleotide identity). We achieved this resolution through multilocus phylogeny, genome-wide gene profiling, and single nucleotide polymorphism (SNP)-based population differentiation analyses of 30 *Bathymodiolus* hybrid and parental individuals collected in 1997 and 2001 at the Broken Spur vent field.

## Materials and methods

A detailed description of samples (Supplementary Table S1) and methods is available in the Supplementary Information and an overview of the analyses of SOX symbionts used in this study is provided in Supplementary Table S2. Data files and scripts used for the analyses can be found in the GitHub repository (https://github.com/muecker/Symbionts_in_a_mussel_hybrid_zone).

Broken Spur parental mussels (13 *B. puteoserpentis*) and hybrids (17 F2–F4 generation hybrids, see Supplement) were identified as described previously [20, 29] (no parental *B. azoricus* were found at Broken Spur). Briefly, mussels were genotyped based on 18 species-diagnostic markers and identified as parental or hybrid mussels using bioinformatic analyses of population structure, admixture and introgression (Supplementary Table S3). After DNA extraction and sequencing, we assembled metagenomes per mussel individual from Illumina short-read sequences. Metagenome-assembled genomes (MAGs) of the SOX symbionts from each mussel individual were binned (for statistics of symbiont MAGs, see Supplementary Table S4), representing the consensus of all SOX symbiont strains in each host individual.

To evaluate genetic differences between symbionts from the northern MAR at the level of bacterial subspecies (sensu Van Rossum et al. [28], here above 97% average nucleotide identity), we used 171 single-copy, gammaproteobacterial marker genes for phylogenomic analysis of the SOX symbiont MAGs and their closest symbiotic relatives, e.g., symbionts of *B. azoricus* from vents north of Broken Spur and *B. puteoserpentis* mussels from vents south of Broken Spur, and free-living relatives (see Supplementary Table S5). To understand which factors affect symbiont composition on the strain level at the northern MAR, we assessed the influence of geographic distance, host species, vent type (basaltic versus ultramafic rock) and depth on SOX symbiont allele frequencies using redundancy analysis (RDA). We analysed Broken Spur symbiont MAGs at the genome-wide level by comparing their average nucleotide identities (ANI) to resolve differences on the subspecies level. To resolve strain-level differences between SOX symbionts from Broken Spur, we analysed pairwise *F*_ST_ values based on SNPs in 2496 orthologous genes from Broken Spur SOX symbiont MAGs. To identify genes that differed between hybrid and parental symbiont populations, we analysed the presence/absence and differential abundance of these orthologues, and further investigated pairwise *F*_ST_ values of all 2496 orthologous genes.

## Results and discussion

**Fig. 1:**
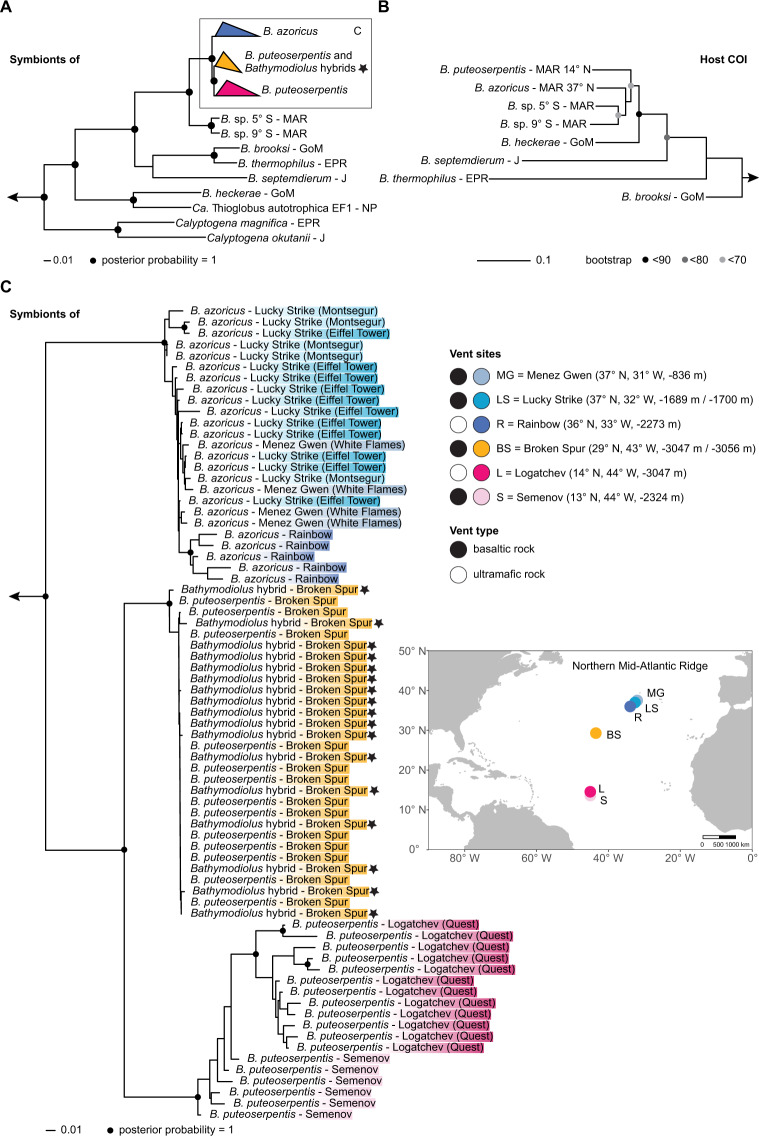
Phylogenetic relationships of *Bathymodiolus* SOX symbionts and their mussel hosts. **A** Overview tree based on 171 single-copy marker genes. The tree was reconstructed based on a 36,949 bp alignment using the LG + F + R6 amino acid model and 1000 samples for ultrafast bootstrap with IQ-TREE. The *Bathymodiolus* SOX symbionts from the northern Mid-Atlantic Ridge (blue, yellow and pink) form a clade within the gammaproteobacterial SUP05 clade. *Thiomicrospira* spp. and *Ca*. T. singularis PS1 were used as outgroups. MAG accessions are listed in Supplementary Table S5. **B** Host phylogeny based on published mitochondrial cytochrome oxidase subunit I (COI) sequences. “*B*.” *childressi* was used as an outgroup. Sequence accessions are listed in the Supplement (“1.3 Reconstruction of *Bathymodiolus* phylogeny”). **C** Zoom in of sequences shown in box in (**A**): Phylogeny of *Bathymodiolus* SOX symbionts from vents on the northern Mid-Atlantic Ridge. Black and white circles indicate the vent type (basaltic or ultramafic rock), colours correspond to vent sites shown in the map. Hybrid individuals from Broken Spur are marked with a black star. *Bathymodiolus* SOX symbionts from the vent sites Clueless (5° S) and Lilliput (9° S) were used as outgroups. *B. Bathymodiolus*, MAR Mid-Atlantic Ridge, GoM Gulf of Mexico, EPR East Pacific Rise, J Japan, NP North Pacific (colour figure online).

Phylogenomic analysis of 171 single-copy genes revealed the presence of two SOX symbiont subspecies, one specific to *B. azoricus* from the more northern vents Menez Gwen, Lucky Strike and Rainbow, and one specific to *B. puteoserpentis* from the vents further south, Logatchev and Semenov (Fig. 1A, C).

This substantiates previous analyses based on sequencing of the 16S rRNA gene and internal transcribed spacer that these two *Bathymodiolus* species harbour different SOX symbiont subspecies of the same bacterial species [21, 25, 30]. Our phylogenomic analyses revealed that all *Bathymodiolus* individuals from Broken Spur harboured a third SOX symbiont subspecies (Fig. 1A, C). This new subspecies is most closely related to the *B. puteoserpentis* SOX symbiont subspecies from mussels collected south of Broken Spur. These two symbiont subspecies form a sister group to the SOX symbiont subspecies of *B. azoricus* collected at vents north of Broken Spur.

**Fig. 2:**
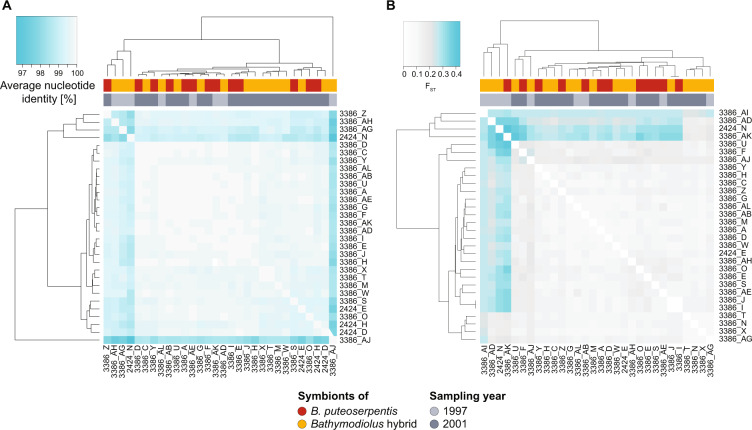
Genome-wide differentiation of *Bathymodiolus* SOX symbionts at Broken Spur. **A** Differentiation based on pairwise average nucleotide identity. **B** Differentiation based on pairwise average *F*_ST_ in 2496 orthologous genes. Colour bars represent host genotypes (red: *B. puteoserpentis*, yellow: hybrids) and the sampling year (light grey: 1997, dark grey: 2001). Turquoise indicates a higher differentiation or more dissimilar genomes. Based on a Mantel test, neither clustering based on ANI (**A**), nor *F*_ST_ (**B**) correlated with host pairwise genetic distances based on 18 SNP markers (**A** *r* = 0.054, *p* = 0.222, **B** *r* = 0.006, *p* = 0.435) or sampling year (**A** *r* = −0.191, *p* = 0.949, **B** *r* = 0.105, *p* = 0.150) (colour figure online).

To evaluate if the SOX symbionts of Broken Spur parental and hybrid *Bathymodiolus* differed, we compared their ANI and estimated genomic differentiation (*F*_ST_) based on ~2500 orthologous genes (for more information on the host, see Supplementary section 2 and Supplementary Table S3). Symbiont ANI values ranged from 96.7 to 99.9% with a median of 99.7%. We found no correlation between symbiont differentiation and the sampling year or genetic differentiation of the mussels (Mantel test of symbiont ANI and *F*_ST_ versus sampling year and host pairwise genetic distances based on 18 SNP markers, Fig. 2). Our analyses of SNPs per individual gene revealed that not even one of the ~2500 orthologous genes had significantly differing *F*_ST_ values (Mann-Whitney *U* test of *F*_ST_ per gene between versus within symbionts of hybrids and parental mussels). Similarly, there was also no significant difference between hybrids and parental individuals in the abundance of symbiont genes (based on a general linear model and Kruskal-Wallace test in ALDEx2 using Benjamini-Hochberg corrected *p* value < 0.05) or their presence/absence. These results indicate that the composition and gene repertoire of SOX symbionts in Broken Spur mussels was highly similar or identical in hybrids and parental *B. puteoserpentis*. A study of SOX symbionts in hybrids of *Bathymodiolus thermophilus* and *Bathymodiolus antarcticus* at 23° S in the eastern Pacific also found that these could not be distinguished from parental mussels, based on PCR analyses of seven bacterial marker genes in five parental and three hybrid individuals [31].

Our results raise the question at what level of genetic divergence between two host species differences in their symbiont communities evolve. *Bathymodiolus brooksi* and *Bathymodiolus heckerae*, which regularly co-occur in the Gulf of Mexico, harbour different symbiont species that are only distantly related to each other (Fig. 1A, B). These two mussel species have an estimated splitting time of 15.4 Mya [17], and are not known to hybridise. More closely related hosts, such as *B. thermophilus* and *B. antarcticus* (estimated splitting time of 2.5–5.3 Mya [32]), and *B. azoricus* and *B. puteoserpentis* (estimated splitting time of 8.4 Mya [17]), produce fertile hybrids [19, 33], and have genetically indistinguishable symbionts in zones where they hybridise. This suggests that specificity at the symbiont species level in these horizontally transmitted symbioses evolves only after extended divergence times of tens of millions of years, during which these hosts become genetically dissimilar enough to evolve specific symbiont selection mechanisms.

**Fig. 3:**
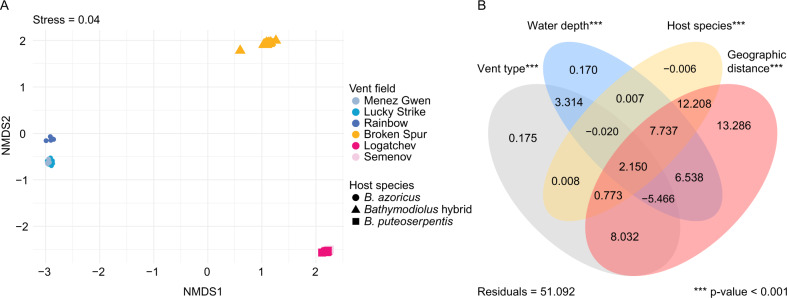
Differentiation of *Bathymodiolus* SOX symbionts at the northern Mid-Atlantic Ridge and the influence of geographic distance, host species and environmental parameters (vent type and water depth). **A** NMDS plot of SOX symbiont allele frequencies show clear separation of the three symbiont subspecies at the northern MAR: *B. azoricus*, *B. puteoserpentis*, and Broken Spur symbiont subspecies. Symbionts from Broken Spur cluster together regardless of the species affiliation of their host (hybrid versus *B. puteoserpentis*). Colours correspond to vent fields, shapes to host species. **B** Variation partitioning of explanatory variables used in the RDA (Supplementary Fig. S3). Values are shown in percent. The variables vent type, water depth, host species, and geographic distance and their interaction effects explained 49% of the total variation, with 13% of the variation solely explained by geographic distance. Negative adjusted *R* values can occur for several reasons, e.g., negative eigenvalues in the model underlying the varpart function in R. *P* values are based on permutation tests with 1000 repetitions.

While *Bathymodiolus* mussels on the northern MAR host the same SOX symbiont species, our phylogenomic analyses revealed clear genetic differentiation in three SOX symbiont subspecies: *B. azoricus*, *B. puteoserpentis* and Broken Spur subspecies (Fig. 1). To better understand the factors that drive this symbiont differentiation, we tested which influence host species, geographic distance, vent type (basaltic versus ultramafic rock) and depth have on symbiont allele frequencies. All variables were highly collinear. For example, the water depth of the vents studied here increases with geographic distance, from 800 m at 37.8° N, to 3050 m at 14.7° N (only the southernmost vent at 13.5° N and 2320 m depth interrupted this pattern). When the four variables were considered individually, geographic distance explained 13% of symbiont differentiation, while the three other variables water depth, host species and vent type each explained 0.2%, 0.0%, and 0.2%, respectively. When interaction effects between the four variables were considered, geographic distance and interactions involving this variable explained 45% of symbiont differentiation, while the three other variables water depth, host species, vent type and the interactions with these explained 14%, 12%, and 9%, respectively (*p* value < 0.001, Fig. 3).

There are at least three explanations for why geographic distance has such a large effect on the SOX symbiont composition of *Bathymodiolus* mussels from the northern MAR. The first is that with increasing geographic distance, environmental differences between vents become larger and these environmental differences affect symbiont composition (genetic isolation by environment versus distance [34]). *Bathymodiolus* mussels acquire their symbionts horizontally from the environment, presumably when the larvae settle on the seafloor [25, 35], and it would be advantageous for the mussels if they selected symbionts that are best adapted to local environmental conditions. We tested the effect of vent type based on one of the key environmental variables at hydrothermal vents, basaltic and ultramafic rock. These two rock types have major effects on the biogeochemistry of vent fluids, including the relative concentrations of the symbiotic energy sources sulphide, methane and hydrogen [36]. However, vent type alone explained only 0.2% of symbiont genetic differentiation, similar to another environmental variable water depth, which also only explained 0.2% of symbiont differentiation. It is therefore unlikely that environmental differences between vents underlie the symbiont population structures we observed in this study.

The second explanation for why geographic distance has such a large effect on the SOX symbiont composition of *Bathymodiolus* mussels is that genetic differences between the hosts increased with geographic distance. However, population genetic analyses of *B. azoricus* and *B. puteoserpentis* from the same vents as in our study indicated no genetic structuring within each of these host species [29]. This indicates that host genetics did not play a major role in structuring the SOX symbiont composition. The third, and most likely explanation is that the free-living pool of SOX symbionts is geographically structured. At Broken Spur, hybrids and *B. puteoserpentis* host genetically indistinguishable symbionts, and these differed from the symbionts of *B. azoricus* and *B. puteoserpentis* from vents to the north and south of Broken Spur. This indicates that in these two closely related host species, geographic location but not host genetics drives the composition of their SOX symbiont communities. Furthermore, the environment, based on vent type, cannot explain why the mussels at Broken Spur had symbionts that are genetically distinct from other vent sites. Broken Spur is basalt-hosted, while the vents to the north are both basalt- (Menez Gwen and Lucky Strike) and ultramafic-hosted (Rainbow). Yet the symbionts from the vents to the north of Broken Spur are more closely related to each other than to the symbionts of Broken Spur mussels (Fig. 1C).

The validity of these three explanations could be tested in future studies by sampling the free-living microbial populations at hydrothermal vents on the MAR. This is, however, not as simple as it sounds because of multiple challenges including obtaining representative samples from the immediate environment of *Bathymodiolus* mussels, collecting environmental data at scales relevant to the microbial population, and characterising the free-living symbiont population at sufficiently high resolution.

Understanding the biogeography of the free-living stages of microbial symbionts and other as yet uncultured microorganisms is currently one of the biggest challenges in microbial ecology. While there is evidence that ‘everything is everywhere, but the environment selects’ [37, 38], there is also increasing data showing that dispersal limitation shapes the biogeography of marine microorganisms [39, 40]. Almost nothing is known about the biogeography of uncultivable marine microorganisms at the subspecies or strain level, as most species are rarely abundant enough to allow phylogenetic analyses at such high resolution. Advances in high-throughput short-read, and particularly long-read sequencing, coupled with bioinformatic methods for revealing genetic structuring of microbial populations, are now providing us with the tools for resolving the intraspecific diversity of environmental microorganisms. Our study highlights the importance of gaining a better understanding of the free-living community of microbial symbionts to disentangle the genetic, environmental, and geographic factors that contribute to the ecological and evolutionary success of animal–microbe associations in which the symbionts are acquired from the environment.

## Supplementary information

Supplementary Information

## Data Availability

Sequence data (metagenomes and symbiont MAGs) are available in the European Nucleotide Archive (ENA) at EMBL-EBI under project accession number PRJEB36976. The data, together with their metadata, were deposited using the data brokerage service of the German Federation for Biological Data (GFBio [41]), with the standard information on sequence data provided as recommended [42].

## Code availability

Additional data files and scripts used in this study are available in the GitHub repository (https://github.com/muecker/Symbionts_in_a_mussel_hybrid_zone).
