# Supplementary material for: Deep-sea mussels from a hybrid zone on the Mid-Atlantic Ridge host genetically indistinguishable symbionts
Source: ISME J. 2021 May 10;15(10):3076–83. doi: 10.1038/s41396-021-00927-9 (PMC8443746; doi:10.1038/s41396-021-00927-9)
Supplement: Supplementary file 1 — Supplementary Information [file 41396_2021_927_MOESM1_ESM.docx]

Supplementary Information

Deep-sea mussels from a hybrid zone on the Mid-Atlantic Ridge host genetically indistinguishable symbionts

Merle Ücker^1,2^, Rebecca Ansorge^1,3^, Yui Sato^1^, Lizbeth Sayavedra^1,3^, Corinna Breusing^4^, Nicole Dubilier^1,2,*^

^1^Max Planck Institute for Marine Microbiology, Bremen, Germany

^2^MARUM – Center for Marine Environmental Sciences of the University of Bremen, Bremen, Germany

^3^Quadram Institute Bioscience, Norwich, Norfolk, United Kingdom

^4^University of Rhode Island, Graduate School of Oceanography, Narragansett, RI, United States of America

*Corresponding author

# Supplementary materials & methods

## Sampling, DNA extraction and metagenomic sequencing

Mussels were sampled at Broken Spur (29°10.0’N, 43°10.0’W at 3045 – 3056 m water depth) with the deep-sea submersible Alvin during the Atlantis cruises AT-03/03 (1997) and AT-05/03 (2001). Upon recovery on board, small mussels (< 25 mm) were frozen whole at -70°C, while larger mussels were dissected before freezing at -70°C [1]. An overview map of sites analysed in this study was plotted with RStudio v1.3.959 using R v3.6.3 and the packages rnaturalearth v0.1.0, legendMap v1.0 and ggplot2 v3.3.0 [2–5].

Genomic DNA was extracted from gill (metagenomic libraries 3386-A~AL) or a combination of gill, mantel and digestive tissue (called mixed tissue hereafter, metagenomic libraries 2424-A~O) depending on sample availability. DNA extractions were performed with either the AllPrep DNA/RNA/Protein MiniKit (Qiagen, Hilden, Germany) or DNAeasy Blood & Tissue kit (Qiagen, Hilden, Germany) according to the manufacturer’s protocols with the following modifications: Prior to extraction, frozen sample pieces (5-10 mm) were homogenised by bead beating in MP Biomedicals Lysing Matrix B using an MP Biomedicals FastPrep-24 (Thermo Fisher Scientific, Waltham, USA) for 30 s at 6.5 m/s. In the elution step, samples were incubated for 10 min at room temperature before centrifugation. Volumes of eluent were halved, and elution was repeated with the first eluate to maximise DNA yields. Metagenomic libraries were generated with the Nextera DNA Flex Library Prep Kit (Illumina, San Diego, CA, USA) and the Illumina TruSeq DNA Samples Prep Kit (BioLABS, Frankfurt, Germany). Library preparation and sequencing of 150 bp paired-end metagenomic reads were performed by the Max Planck-Genome-centre Cologne, Germany (<https://mpgc.mpipz.mpg.de/home/>) on HiSeq 2500 or 3000 machines. Details of sampling, DNA extraction and sequencing of all samples used in this study are summarised in Supplementary Table S 1.

## Identification of hybrid host individuals

Mussels were genotyped based on 18 species-diagnostic single-nucleotide polymorphism (SNP) markers and identified as hybrid or parental species with subsequent bioinformatic analyses using 1) STRUCTURE v2.3.4 with strauto v1.0 and CLUMPAK (<http://clumpak.tau.ac.il/>), 2) introgress v1.22 in RStudio, and 3) NEWHYBRIDS v1.1 [6–13]. The analysis is based on the method developed in [14,15]. Results of all three programmes are shown in Supplementary Table S 3. Classification based on introgress was used as the basis for analyses of mussel symbionts, as it had low misidentification rates for hybrid and parental species as reported in [15]. Since not all programmes supported the classification of backcrosses and their identification was less reliable than for hybrid and parental species in [15], these samples were excluded from further analyses.

## Reconstruction of *Bathymodiolus* phylogeny

Sequences of the mitochondrial marker gene cytochrome c oxidase subunit I or full mitochondrial genomes were downloaded from NCBI (database accessed 2020-02-11) for *B. azoricus* (LN833437), *B. brooksi* (KU597634), *B. heckerae* (KU659139), *B. puteoserpentis* (KU597632), *B.* sp. Lilliput (LN833440), *B.* sp. Clueless (LT674164), *B. septemdierum* (AP014562), *“B.” childressi* (ANY30357) and *B. thermophilus* (MK721544) [16]. We aligned the sequences with MUSCLE v3.8.31 [17,18], and reconstructed a phylogenetic tree using IQTREE v1.6.9 with 1000 samples for ultrafast bootstrap and the mtZoa model, which was selected as the best model by Model Finder based on the Bayesian Information Criterion [19–22]. The tree was visualised with iTol v5.5 [23] and edited with Adobe Illustrator 2020 [24].

## Metagenome assembly and symbiont binning

Metagenomic reads were adapter-trimmed and quality-filtered to a PHRED score of 2 with BBDuk, merged with BBMerge, and error-corrected and normalized to 80x average coverage with BBNorm from BBTools v37.28 [25]. Merged and unmerged reads were assembled with Megahit v1.0.3 using a maximum k-mer size of 127 [26,27]. Initial metagenome-assembled genomes (MAGs) were obtained with Metabat2 v2.10.2 automated binning, after mapping with BBMap, and sorting of bam files with samtools v1.9 [28,29]. MAGs were identified based on small subunit ribosomal RNA gene sequences (SSUs, detected by barrnap v0.6 and classified with vsearch v2.6.2 against the SILVA SSU database v132) and other taxonomic marker genes (detected by Amphora2), through visualisation with gbtools v2.6.0 in RStudio [30–35]. This metagenomic workflow was initially performed separately for gill and mixed tissue. If both sample types were available for the same mussel individual, we confirmed that their symbiont MAGs were identical based on GC content, coverage, and average nucleotide identity (ANI) before pooling the reads and repeating the workflow to increase symbiont coverage. Similarly, if Metabat2 split the sulphur-oxidising (SOX) symbiont sequences into multiple bins, these were pooled after assessment of their GC content, coverage and ANI. Completeness, contamination and strain heterogeneity of MAGs were estimated throughout the binning process with CheckM v1.0.17 based on 280 gammaproteobacterial marker genes [36–39]. Due to the presence of multiple strains in the symbiont population [40], we expected duplicates of marker genes with highly similar sequences, such as the ones reported by CheckM as strain heterogeneity. We therefore corrected contamination rates and calculated the contamination that could not be attributed to strain heterogeneity.

For MAGs with a completeness below 90 %, an additional step of assembly and binning was performed to yield MAGs with higher completeness. In such cases, the incomplete bin was used as a reference for mapping to recruit symbiont reads for assembly with SPAdes v3.12.0 [41]. These draft genome assemblies were manually binned in Bandage v0.8.1 based on sequence nodes connected in the assembly graph [42].

Additional statistics of symbiont MAGs were calculated using the stats.sh script of BBTools (Supplementary Table S 4). Read coverage of symbiont MAGs was estimated with samtools after mapping raw reads against MAGs with BBMap. High-quality symbiont MAGs with a completeness above 90% and a contamination below 5% (after correction for strain heterogeneity) were used for further analysis with one exception: The MAG of library 3386_H was 89 % complete, only <1 % less than the cutoff, but had no contamination.

## Analyses of SOX symbionts based on symbiont MAGs

All analyses of SOX symbionts conducted in this study, their input data and the level of resolution are summarised in Supplementary Table S 2.

### Phylogenomic analysis of SOX symbionts

To investigate the phylogeny of SOX symbionts from mussels in Broken Spur, we constructed a phylogenomic tree with 171 gammaproteobacterial, single-copy marker genes from the SOX MAGs as well as closely-related symbiotic and free-living bacteria. Accession numbers and publications for all reference MAGs/genomes used in the analysis are listed in Supplementary Table S 5.

A protein alignment of the 171 marker genes (Data file: Phylogenomics_NMAR.fasta, available at <https://github.com/muecker/Symbionts_in_a_mussel_hybrid_zone>) was obtained with GToTree v1.4.11 [17,18,38,39,43,44]. The alignment was visually inspected in Geneious v11.1.5 [45]. One gene sequence (of sample 1586K) had a high proportion of mismachtes to all other sequences and was identified as contamination by blasting against the NCBI database. This sequence was removed from the alignment. Using the LG+F+R6 amino acid model ([46], best model according to Model Finder), and 1000 samples for ultrafast bootstrap, we reconstructed a phylogenomic tree of SOX symbionts and their closest relatives with IQ-TREE v1.6.7.1, and edited it with iTol and Adobe Illustrator.

Correlation between symbiont F_ST_ and geographic distance was tested using the Mantel test. The multiple sequence alignment was imported into R with the read.alignment function of R package seqinr v3.4-5 and transformed into a genind object using the alignment2genind function of adegenet v2.1.1 [47,48]. We caluclated the pairwise F_ST_ of symbionts between all locations on the northern MAR using pairwise.fst of R package hierfstat v0.04-33 [49]. Geographic distances between vent sites were calculated based on the coordinates using <https://www.movable-type.co.uk/scripts/latlong.html>, and transformed into a dist object using the R function dist. To account for the geographic subdivision of the host species that can also cause patterns similar to isolation-by-distance [50], we performed a stratified Mantel test using the mantel function of R package vegan v2.5-5, and the host species groups as strata (mantel(FST, geo_distances,strata=groups_NMAR) [51]. After a statistical significant result, the F_ST_ values between symbionts from different sites were plotted against their geographic distances for visual inspection.

### Average nucleotide identity of SOX symbionts

To analyse how similar symbiont MAGs from Broken Spur were to each other, we analysed their pairwise ANI values of the aligned fraction (0.48 – 0.99%) with fastANI v1.1 [52] (Data file: Average_nucleotide_identity_SOX_symbionts_Broken_Spur.csv). Samples were clustered and represented based on their average ANI values in a heatmap with dendrogram, generated in RStudio using the packages gplots v3.0.1.1 and maditr v0.6.2 [53,54].

Correlation of SOX symbiont ANI values and sampling year was tested with a Mantel test (mantel function of R package vegan, 5039 permutations) using the Spearman’s rank correlation coefficient after transforming sampling year information into euclidean distances. To test the correlation between SOX symbiont ANI values and host genetics, pairwise genetic distances between host individuals were calculated based on 18 species-diagnostic SNP markers (see “1.2. Identification of hybrid host individuals”). Host SNP markers were imported to RStudio and converted into a dataframe using the read.structure and genind2df functions of the package adegenet. We subsequently calculated pairwise genetic distances between individuals with the dist.gene function of R package ape v5.3 [55]. Correlation was tested as described above for ANI values vs. sampling year.

## Analyses of symbiont population based on single-nucleotide polymorphisms

Recent advances in whole-(meta)genome approaches have increased resolution and sensitivity of analyses, and advanced our knowledge on strain diversity in deep-sea mussel symbiont populations [40,56,57]. We therefore performed genome-wide SNP analyses of SOX symbionts based on 2496 orthologous genes to investigate symbiont population differentiation between different mussel individuals from Broken Spur.

We used a gene catalogue of 3204 orthologues (see below) as a reference for SNP identification. The catalogue was annotated using prokka v1.1, resulting in 2496 genes with annotation (including hypothetical proteins) [58]. Genes without any annotation, mostly short (<300 bp), probably fragmented genes, were excluded from the analysis. SNP calling was performed as described in [40] using scripts available at <https://github.com/rbcan/MARsym_paper> with a few adjustments to newer software versions. In summary, raw reads were adapter trimmed and quality filtered to a PHRED score of 20 and subsequently mapped to the reference with a minimum identitiy of 95 % using BBMap. We realigned reads around indels and downsampled to an average coverage of 70x. Samples that did not meet the coverage threshold were excluded from the analysis. The steps above were performed with samtools, Picard tools v1.1.02 and the Genome Analysis Toolkit (GATK) v3.7-0 [29,59,60]. SNPs were called with GATK HaplotypeCaller, and unreliable SNPs were filtered with GATK VariantFiltration (settings: QD < 2; FS > 60; MQ < 40, MQRankSum < -20, ReadPosRankSum < -8). Spearman’s rank correlation coefficient was calculated in RStudio using the R function cor to test for correlation of SNP density (#SNPs/kb) with shell size. The fixation index F_ST_ was calculated for each gene with a script (<https://github.com/deropi/BathyBrooksiSymbionts/tree/master/Population_structure_analyses>) previously used in [57], and averaged per host individual (Data file: Pairwise_mean_FST_SOX_symbionts_Broken_Spur.csv). Mean pairwise F_ST_ values were plotted in a heatmap, and correlation between F_ST_ and sampling year and F_ST_ and host genotype was tested as described above for ANI values.

## Analysis of differences in gene repertoire between symbionts from hybrids and parental species

### Gene presence/absence and abundance analyses

To examine whether there are differences in the gene repertoire of the symbiont populations between hybrid and parental mussels, we analysed the presence/absence of genes specific to either group of mussels and their relative abundances. We annotated all MAGs with prokka and clustered orthologues with GET_HOMOLOGUES v3.2.3 using the OrthoMCL algorithm [39,61–69], resulting in a gene catalogue of 3204 orthologues (Data file: Orthologue_gene_catalogue_OMCL_SOX_symbionts_Broken_Spur.fasta; also used in SNP-identification above). Using the parse_pangenome_matrix.pl script of GET_HOMOLOGUES, we tested for genes that were present in at least 90 % of symbiont genomes from *B. puteoserpentis* and absent in at least 90 % of symbiont genomes from hybrids, and vice versa.

To further analyse gene abundances, raw reads of all libraries were mapped to the orthologous gene catalogue with BBMap and downsampled to 70x coverage with samtools. The fasta sequences were extracted from downsampled bam files using samtools and pseudoaligned to the catalogue using kallisto v0.46.0 [25,70]. The gene coverage was estimated using the abundance_estimates_to_matrix.pl script of Trinity v2.5.1 [71,72] (Data file: Gene_counts_kallisto_SOX_symbionts_Broken_Spur.matrix).

To account for the compositionality of the data, the gene abundances were statistically evaluated using ALDEx2 v1.16.0 and data.table v1.12.2 in RStudio [2,73–76]. We used the aldex.clr module to prepare the data using host categories (hybrid or *B. puteoserpentis*) as condition. With the aldex.kw command, we ran a general linear model and a Kruskal Wallace test for one way ANOVA.

### Analysis of gene differentiation between symbionts from hybrids and parentals

We analysed population differentiation (F_ST_) based on SNP frequencies in 2496 orthologue genes to find genes that have higher differentiation ‘between’ symbionts of hybrids and parental species than variations ‘within’ symbionts of the same host category (hybrids or *B. puteoserpentis*, Supplementary Figure S 1). F_ST_ values were acquired as described above (1.6 “Analyses of symbiont population based on single-nucleotide polymorphisms”) and reformatted for ‘between’ versus ‘within’ statistical comparisons (Data file: Per_gene_FST_SOX_symbionts_Broken_Spur.zip). We used a Mann–Whitney U test in RStudio to test the null hypothesis that there is no significant difference between F_ST_ of genes in the ‘between’ (hybrids versus parental species) and the ‘within’ (hybrids versus hybrids, parental versus parental species) categories. To reduce false discovery rates, the test was repeated with a dataset of random F_ST_ values. More genes with p-value <0.05 were detected in the random dataset than in the actual data, and no p-values of the real dataset were below those from the random dataset.

This indicates that all genes detected as significant (p<0.05) for the real data can be attributed to type I error, and that there was no gene more differentiated between symbionts of hybrid and parental mussels than within the same host category.


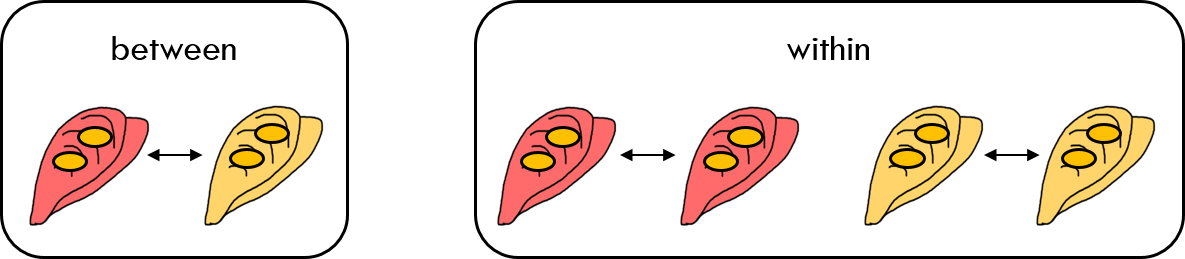


**Supplementary Figure S 1 | Categories for per gene F_ST_ analysis.** Between: Comparison of SOX symbionts from *B. puteoserpentis* (red) and hybrid mussels (yellow). Within: Comparison among SOX symbionts from *B. puteoserpentis* and among SOX symbionts from hybrid mussels.

## Redundancy analysis of SOX symbiont allele frequencies from *Bathymodiolus* mussels along the northern Mid-Atlantic Ridge

Redundancy analysis (RDA) allows to eliminate redundant information in genetic data and its associations with environmental variables, and to assess the proportion of variation explained by these environmental variables [77]. We performed a RDA in RStudio using the vegan package to test how much of the variation in symbiont allele frequencies can be explained by geographic distance, vent type (basaltic versus ultramafic rock), the associated host species and depth. In hydrothermal systems, rock type plays a central in role in determining biogeochemical conditions, including pH and the energy sources available for chemosynthetic microorganisms [78], which is why we chose this environmental parameter, which we called vent type. Depth and vent type were retrieved from the InterRidge Vents Database v3.4 (<https://vents-data.interridge.org/>, accessed 2020-06-15). As a reference for SNP analysis, we constructed a gene catalogue based on all SOX symbiont MAGs from the northern MAR (Data file: Orthologue_gene_catalogue_OMCL_SOX_symbionts_NMAR.fasta) using the workflow described above (1.7.1 “Gene presence/absence and abundance analyses”). To obtain allele frequencies, we performed a SNP analysis based on the gene catalogue of all SOX symbionts from the northern MAR as described above (1.6 “Analyses of symbiont population based on single-nucleotide polymorphisms”). We extracted the AD (read depth per allele) and DP (read depth) field from VCF files using GATK’s VariantToTable tool, divided AD by DP to obtain allele frequencies per sample and merged the individual tables using join on the Linux command line (Data file: Allele_frequencies_SOX_symbionts_NMAR.csv). For the RDA, site coordinates were scaled and computed as orthogonal polynomials with R package stats v3.6.3 (function poly) as suggested by [77,79,80]. We performed a forward selection on the polynomials with the ordistep function of R package vegan, ran the RDA with all variables and calculated an adjusted R². We assessed the significance of the RDA, the individual axes and the explanatory variables with the vegan package function anova.cca using 1000 permutations. To explore how much variation could be explained by each explanatory variable, we performed a variation partitioning using the varpart function of vegan and plotted it with R base function plot. The RDA triplot was plotted using the ggord package v1.1.4 [81]. For an overview visualisation of the allele frequencies, we calculated a NMDS using the metaMDS function of vegan and plotted it with ggplot2. All figures were modified with Adobe Illustrator.

# Supplementary results & discussion

## *B. puteoserpentis* and hybrid individuals identified in Broken Spur

We genotyped mussels from Broken Spur and identified *B. puteoserpentis* and hybrid individuals. *B. azoricus* mussels were not detected in all methods used, except for one mussel (3676-15/3386_N) that was identified as *B. azoricus* by NEWHYBRIDS. However, this result was not supported by the two other programmes, suggesting that the mussel is more likely a hybrid.

The absence of *B. azoricus* in Broken Spur could be due to bathymetric limitation, as *B. azoricus* usually occurs at shallower depths. Another possible explanation is that the actual hybrid zone might be further north as suggested by [15]. Lastly, it cannot be ruled out that *B. azoricus* mussels were not found during sampling as the number of mussels collected was limited and their distribution quite patchy at Broken Spur.

All hybrids identified by INTROGRESS were in the F2 to F4 generation indicating that the hybrids are fertile. Although the exact status of backcrosses, especially which generations of backcrosses are actually present, was uncertain, multiple mussels were identified as backcrosses by NEWHYBRIDS and INTROGRESS. Together with the admixture values reported by STRUCTURE, this suggests that there is still gene flow between hybrids and the populations of parental species.

## Future studies on *Bathymodiolus* hybrids

Hybrids from Broken Spur were clearly able to successfully reproduce given the presence of F2 – F4 hybrids, but we have no information on hybrid performance and fitness. In lab-held organisms, fitness of hybrids can be assessed by various measurements, e.g. comparisons of offspring survival rates or developmental times [82]. To study these parameters in *Bathymodiolus* mussels, the mussels would have to be maintained in aquaria until they are ready to spawn, which may occur only once a year in January in *B. azoricus* [83]. After spawning, eggs could be collected for counting and genotype determination. However, embryo development in aquaria-held mussels has not yet succeeded, as previous attempts failed because development was abnormal or stopped at the 4-cell stage [83,84]. An alternative option, equally challenging, would be to collect *Bathymodiolus* mussels from a hybrid zone, e.g. Broken Spur on the MAR or the vents at 23°S on the East Pacific Rise [85], and determine their genotype prior to cultivation (e.g. by removing hemolymph from their adductor muscles). To ensure reproducible results, enough replicates would be needed, which is generally challenging for most deep-sea species. In model organisms that can be easily cultured in the laboratory, such studies can be performed more easily. Studies in *Nasonia* or *Drosophila* pointed towards poor hybrid performance, i.e. high lethality and sterility of hybrid individuals [86–89].

## No difference in gene abundances between symbionts from hybrids and parental species

We analysed 3204 orthologous genes to detect genes that are exclusive to either symbionts of hybrid or symbionts of *B. puteoserpentis*. GET_HOMOLOGUES detected none of such genes, even with lower stringency (presence in >90 % in one and <90 % in the other group).

When comparing gene abundances between symbionts from hybrid and *B. puteoserpentis* mussels using the statistical analysis with ALDEx2, no genes were significantly different in their abundances (Benjamini-Hochberg corrected p-value < 0.05). Functional variation has previously been shown to occur among symbiont populations from different vents along the MAR [40]. However, the gene repertoire of SOX symbiont populations within Broken Spur did not vary according to host genotype, indicating that hybrids and parental species do not select their symbionts based on different functions.

## No correlation of SNPs/kb with mussel shell size

Picazo et al. (2019) previously detected lower strain diversity in large (146–241 mm) compared to medium-sized (72–141 mm) mussels in the Gulf of Mexico that might be explained by self-infection and slower symbiont uptake in older mussels [57]. We did not detect any correlation of SNPs/kb with shell size, which might be due to the relatively limited size range of the analysed mussels (24–133 mm).

## Isolation-by-distance of *Bathymodiolus* SOX symbiont subspecies at the northern MAR

Phylogenomic analysis of 171 gammaproteobacterial marker genes revealed that symbiont genetic variation (F_ST_ based on the amino acid alignment) was positively correlated with geographic distance (r = 0.7471, p = 0.035). However, a gradual genetic change along a geographic gradient as would be expected under an evolutionary isolation-by-distance (IBD) model [90] could not be observed (Supplementary Figure S 2). Mantel tests are often used to test for isolation-by-distance which is why we included this analysis here. However, its use has been discouraged [91]. We therefore used a redundancy analysis in this study (Figure 3, Supplementary Figure S 3, Supplement 1.8 “Redundancy analysis of SOX symbiont allele frequencies from *Bathymodiolus* mussels along the northern Mid-Atlantic Ridge” and main text).


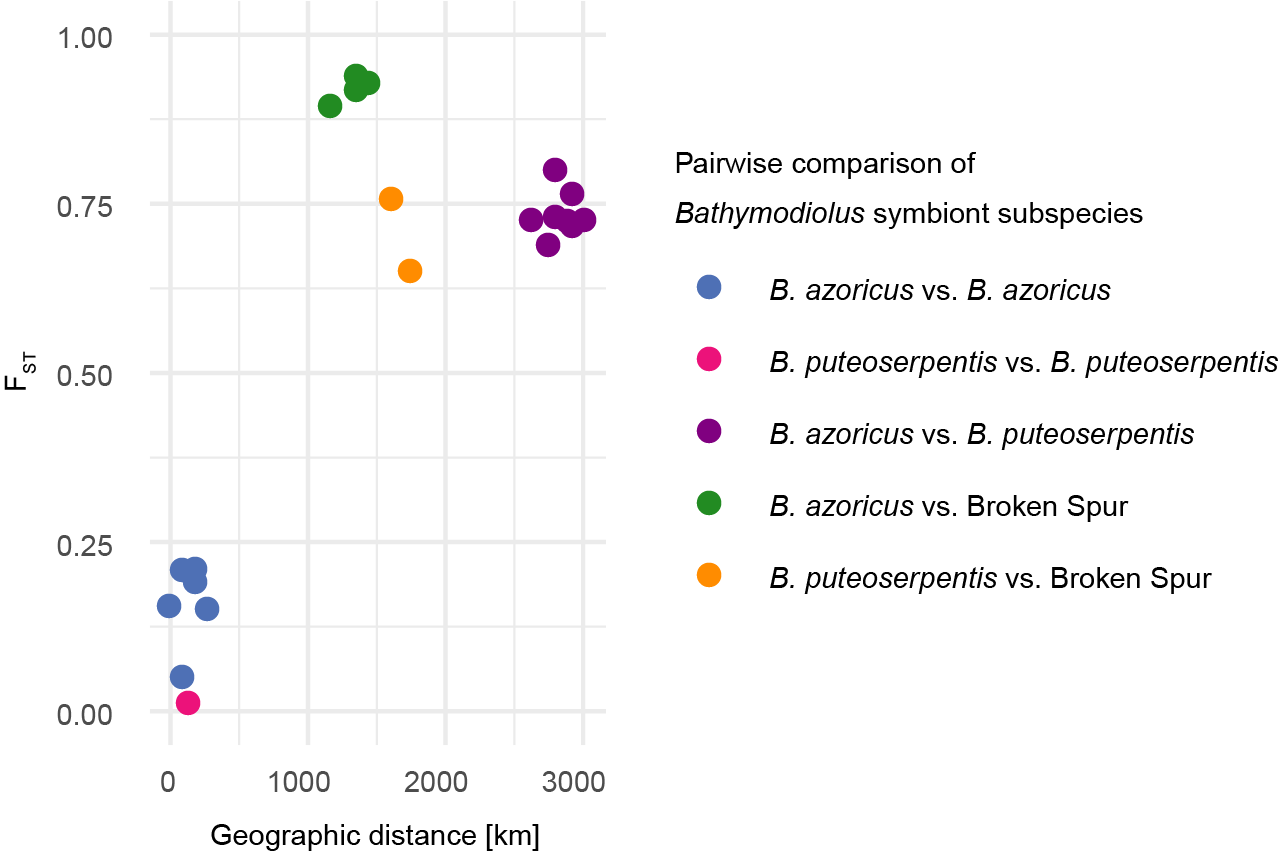


**Supplementary Figure S 2 | Relation of F_ST_ and geographic distance between *Bathymodiolus* SOX symbiont populations from different vent fields along the northern MAR.** Displayed F_ST_ values are averaged pairwise F_ST_ between symbionts from all mussels at a vent field. Each dot represents one pairwise comparison between two sites, comparisons of a site with itself are not shown. Colours correspond to the comparisons of the different symbiont subspecies (*B. azoricus* type, present at Menez Gwen – White Flames, Lucky Strike – Montsegur, Lucky Strike – Eiffel Tower and Rainbow; *B. puteoserpentis* type, present at Logatchev Quest and Semenov; Broken Spur type, present at Broken Spur).


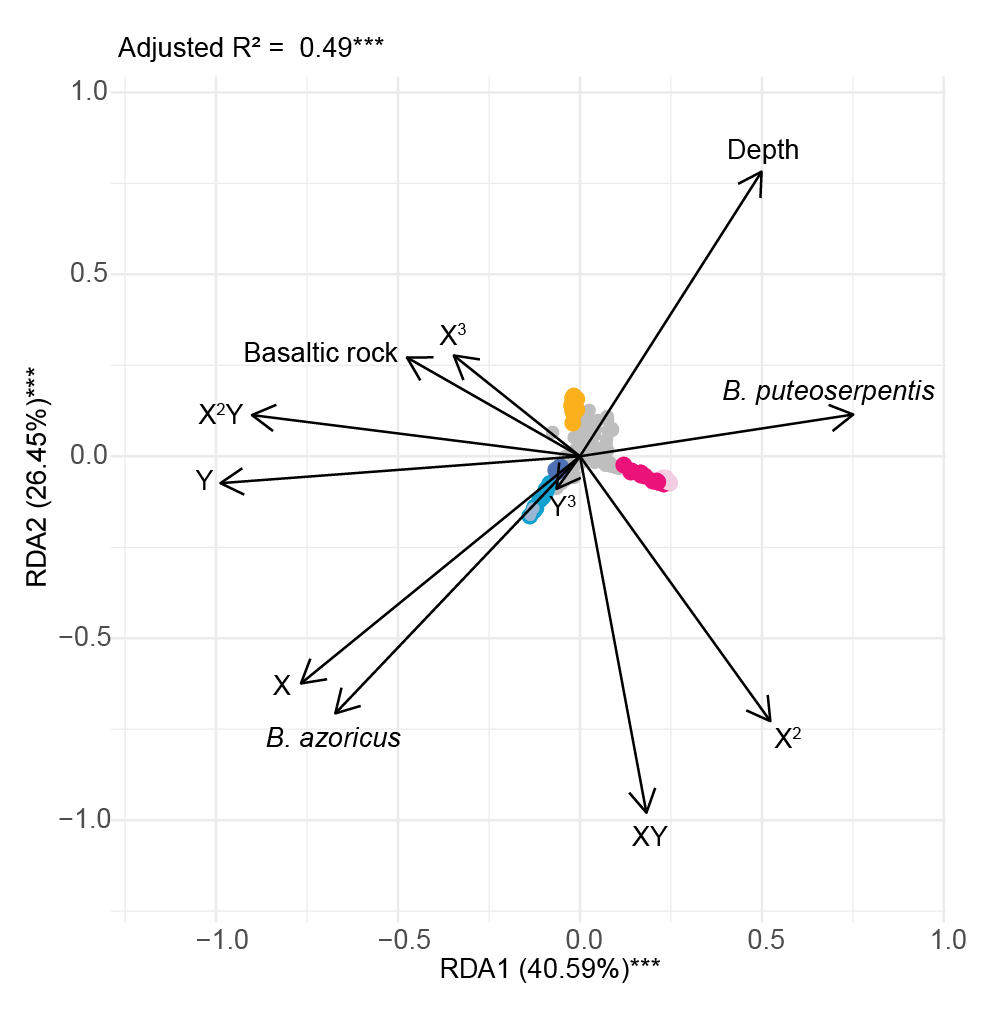


**Supplementary Figure S 3 | Influence of geographic distance, host species and environmental parameters on differentiation of *Bathymodiolus* SOX symbionts at the northern MAR.** Redundancy analysis triplot (scaling 2, wa scores) showing the influence of geographic distance (forward selected variables X, Y, X^2^, X^3^, X^2^Y, Y^3^ represent orthogonal polynomials of latitude and longitude), host species (*B. azoricus* and *B. puteoserpentis*), the vent type (only basaltic rock displayed) and water depth on symbiont allele frequencies. *** p-value < 0.001. P-values are based on permutation tests with 1000 repetitions.

**Supplementary Table S 1 | Sample overview.** ID consists of dive number and mussel ID. Shell length (size) is listed when data were available according to cruise material in <http://dlacruisedata.whoi.edu/AT/AT003L03/> (accessed 2019-05-29). AllPrep: AllPrep DNA/RNA/Protein MiniKit (Qiagen); DNAeasy: DNAeasy Blood & Tissue kit (Qiagen), Nex: Nextera DNA Flex Library Prep Kit (Illumina); TruSeq: Illumina TruSeq DNA Samples Prep Kit (BioLABS).

| **Cruise** | **ID** | **Latitude** | **Longitude** | **Depth [m]** | **Sampling date** | **Material** | **DNA extraction** | **Libprep** | **Sequencing** | **Library** | **Size [mm]** |
| --- | --- | --- | --- | --- | --- | --- | --- | --- | --- | --- | --- |
| AT_05/03 | 3676-1 | 29.1672 | -43.1742 | 3045 | July 19, 2001 | gill | AllPrep | Nex | HiSeq 3000 | 3386_A | 119.8 |
| AT_05/03 | 3676-2 | 29.1672 | -43.1742 | 3045 | July 19, 2001 | gill | AllPrep | Nex | HiSeq 3000 | 3386_B | 113.8 |
| AT_05/03 | 3676-3 | 29.1672 | -43.1742 | 3045 | July 19, 2001 | gill | AllPrep | Nex | HiSeq 3000 | 3386_C | 112.2 |
| AT_05/03 | 3676-4 | 29.1672 | -43.1742 | 3045 | July 19, 2001 | gill | AllPrep | Nex | HiSeq 3000 | 3386_D | 116.3 |
| AT_05/03 | 3676-5 | 29.1672 | -43.1742 | 3045 | July 19, 2001 | gill | AllPrep | Nex | HiSeq 3000 | 3386_E | 119.1 |
| AT_05/03 | 3676-6 | 29.1672 | -43.1742 | 3045 | July 19, 2001 | gill | AllPrep | Nex | HiSeq 3000 | 3386_F | 114.6 |
| AT_05/03 | 3676-7 | 29.1672 | -43.1742 | 3045 | July 19, 2001 | gill | AllPrep | Nex | HiSeq 3000 | 3386_K | 107.6 |
| AT_05/03 | 3676-8 | 29.1672 | -43.1742 | 3045 | July 19, 2001 | gill | AllPrep | Nex | HiSeq 3000 | 3386_G | 103.6 |
| AT_05/03 | 3676-9 | 29.1672 | -43.1742 | 3045 | July 19, 2001 | gill | AllPrep | Nex | HiSeq 3000 | 3386_H | 75 |
| AT_05/03 | 3676-10 | 29.1672 | -43.1742 | 3045 | July 19, 2001 | gill | AllPrep | Nex | HiSeq 3000 | 3386_I | 122.9 |
| AT_05/03 | 3676-11 | 29.1672 | -43.1742 | 3045 | July 19, 2001 | mixed & gill | DNAeasy & AllPrep | TruSeq, Nex | HiSeq 2500 & 3000 | 3386_J, 2424_A | 101.9 |
| AT_05/03 | 3676-12 | 29.1672 | -43.1742 | 3045 | July 19, 2001 | gill | AllPrep | Nex | HiSeq 3000 | 3386_L | 114.8 |
| AT_05/03 | 3676-14 | 29.1672 | -43.1742 | 3045 | July 19, 2001 | mixed & gill | DNAeasy & AllPrep | TruSeq & Nex | HiSeq 2500 & 3000 | 3386_M, 2424_B | 81.4 |
| AT_05/03 | 3676-15 | 29.1672 | -43.1742 | 3045 | July 19, 2001 | mixed & gill | DNAeasy & AllPrep | TruSeq & Nex | HiSeq 2500 & 3000 | 3386_N, 2424_C | 92.9 |
| AT_05/03 | 3676-16 | 29.1672 | -43.1742 | 3045 | July 19, 2001 | mixed & gill | DNAeasy & AllPrep | TruSeq & Nex | HiSeq 2500 & 3000 | 3386_O, 2424_F | 12.2 |
| AT_05/03 | 3676-17 | 29.1672 | -43.1742 | 3045 | July 19, 2001 | gill | AllPrep | Nex | HiSeq 3000 | 3386_P | 110.2 |
| AT_05/03 | 3676-18 | 29.1672 | -43.1742 | 3045 | July 19, 2001 | gill | AllPrep | Nex | HiSeq 3000 | 3386_Q | 89.9 |
| AT_05/03 | 3676-19 | 29.1672 | -43.1742 | 3045 | July 19, 2001 | gill | AllPrep | Nex | HiSeq 3000 | 3386_R | 64.7 |
| AT_05/03 | 3676-20 | 29.1672 | -43.1742 | 3045 | July 19, 2001 | mixed & gill | DNAeasy & AllPrep | TruSeq & Nex | HiSeq 2500 & 3000 | 3386_S, 2424_G | 93.6 |
| AT_05/03 | 3676-21 | 29.1672 | -43.1742 | 3045 | July 19, 2001 | gill | AllPrep | Nex | HiSeq 3000 | 3386_T | 93.2 |
| AT_05/03 | 3676-22 | 29.1672 | -43.1742 | 3045 | July 19, 2001 | gill | AllPrep | Nex | HiSeq 3000 | 3386_U | 81.5 |
| AT_05/03 | 3676-26 | 29.1672 | -43.1742 | 3045 | July 19, 2001 | mixed | DNAeasy | TruSeq | HiSeq 2500 | 2424_D | 31.7 |
| AT_05/03 | 3676-27 | 29.1672 | -43.1742 | 3045 | July 19, 2001 | gill | AllPrep | Nex | HiSeq 3000 | 3386_V | 65.4 |
| AT_05/03 | 3676-28 | 29.1672 | -43.1742 | 3045 | July 19, 2001 | mixed | DNAeasy | TruSeq | HiSeq 2500 | 2424_E | 66.7 |
| AT_05/03 | 3676-29 | 29.1672 | -43.1742 | 3045 | July 19, 2001 | mixed | DNAeasy | TruSeq | HiSeq 2500 | 2424_H | 51.8 |
| AT_05/03 | 3676-30 | 29.1672 | -43.1742 | 3045 | July 19, 2001 | gill | AllPrep | Nex | HiSeq 3000 | 3386_W | 94.4 |
| AT_05/03 | 3676-31 | 29.1672 | -43.1742 | 3045 | July 19, 2001 | gill | AllPrep | Nex | HiSeq 3000 | 3386_X | 64.5 |
| AT_05/03 | 3676-32 | 29.1672 | -43.1742 | 3045 | July 19, 2001 | mixed & gill | DNAeasy & AllPrep | TruSeq & Nex | HiSeq 2500 & 3000 | 3386_Y, 2424_I | 88.8 |
| AT_05/03 | 3676-33 | 29.1672 | -43.1742 | 3045 | July 19, 2001 | mixed & gill | DNAeasy & AllPrep | TruSeq & Nex | HiSeq 2500 & 3000 | 3386_Z, 2424_J | 100.5 |
| AT_05/03 | 3676-37 | 29.1672 | -43.1742 | 3045 | July 19, 2001 | gill | AllPrep | Nex | HiSeq 3000 | 3386_AA | 111.4 |
| AT_05/03 | 3676-38 | 29.1672 | -43.1742 | 3045 | July 19, 2001 | gill | AllPrep | Nex | HiSeq 3000 | 3386_AB | 104.7 |
| AT_03/03 | 3125-1 | 29.1667 | -43.1733 | 3056 | July 17, 1997 | gill | AllPrep | Nex | HiSeq 3000 | 3386_AC |  |
| AT_03/03 | 3125-2 | 29.1667 | -43.1733 | 3056 | July 17, 1997 | gill | AllPrep | Nex | HiSeq 3000 | 3386_AD |  |
| AT_03/03 | 3125-3 | 29.1667 | -43.1733 | 3056 | July 17, 1997 | gill | AllPrep | Nex | HiSeq 3000 | 3386_AE |  |
| AT_03/03 | 3125-4 | 29.1667 | -43.1733 | 3056 | July 17, 1997 | gill | AllPrep | Nex | HiSeq 3000 | 3386_AF |  |
| AT_03/03 | 3125-5 | 29.1667 | -43.1733 | 3056 | July 17, 1997 | mixed & gill | DNAeasy & AllPrep | TruSeq & Nex | HiSeq 2500 & 3000 | 3386_AG, 2424_K |  |
| AT_03/03 | 3125-6 | 29.1667 | -43.1733 | 3056 | July 17, 1997 | mixed & gill | DNAeasy & AllPrep | TruSeq & Nex | HiSeq 2500 & 3000 | 3386_AH, 2424_L |  |
| AT_03/03 | 3125-7 | 29.1667 | -43.1733 | 3056 | July 17, 1997 | mixed & gill | DNAeasy & AllPrep | TruSeq & Nex | HiSeq 2500 & 3000 | 3386_AI, 2424_M |  |
| AT_03/03 | 3125-8 | 29.1667 | -43.1733 | 3056 | July 17, 1997 | mixed | DNAeasy | TruSeq | HiSeq 2500 | 2424_N |  |
| AT_03/03 | 3125-9 | 29.1667 | -43.1733 | 3056 | July 17, 1997 | mixed & gill | DNAeasy & AllPrep | TruSeq & Nex | HiSeq 2500 & 3000 | 3386_AJ, 2424_O |  |
| AT_03/03 | 3125-10 | 29.1667 | -43.1733 | 3056 | July 17, 1997 | gill | AllPrep | Nex | HiSeq 3000 | 3386_AK |  |
| AT_03/03 | 3125-11 | 29.1667 | -43.1733 | 3056 | July 17, 1997 | gill | AllPrep | Nex | HiSeq 3000 | 3386_AL |  |

**Supplementary Table S 2 | Overview of analyses of Bathymodiolus SOX symbionts.**

| **Analysis** | **Input data/reference** | **Level of resolution** |
| --- | --- | --- |
| Phylogenomics | 171 marker genes extracted from NMAR MAGs | symbiont subspecies |
| Average nucleotide identity | Broken Spur MAGs | symbiont subspecies |
| Gene abundances | Mussel metagenomes from Broken Spur mapped against Broken Spur orthologous gene catalogue | symbiont strain |
| SNP analyses | Mussel metagenomes from Broken Spur mapped against Broken Spur orthologous gene catalogue | symbiont strain |
| Redundancy analysis | Mussel metagenomes from NMAR sites mapped against NMAR orthologous gene catalogue | symbiont strain |

**Supplementary Table S 3 | Genotyping results of 42 mussel individuals from Broken Spur based on analyses with NEWHYBRIDS, INTROGRESS and STRUCTURE.** ID: Dive number–mussel ID; Lib: Metagenomic library name; Bazo: *B. azoricus*; BC azo: Backcross between *B. azoricus* and hybrid; FX: Hybrid in generation X; BC put: Backcross between *B. puteoserpentis* and hybrid; Bput: *B. puteoserpentis*; Genotype: NEWHYBRIDS’ genotype category with highest probability.

| **ID** | **Lib** | **NEWHYBRIDS** | | | | | | | | | | | | | **INTROGRESS** | **STRUCTURE** | |
| --- | --- | --- | --- | --- | --- | --- | --- | --- | --- | --- | --- | --- | --- | --- | --- | --- | --- |
|  |  | **Bazo** | **BC1 azo** | **BC2 azo** | **BC3 azo** | **BC4 azo** | **F1** | **F2-4** | **BC1 put** | **BC2 put** | **BC3 put** | **BC4 put** | ***Bput*** | **Genotype** |  | **Bazo** | **Bput** |
| 3676-1 | 3386_A | 0.00 | 0.00 | 0.00 | 0.00 | 0.00 | 0.00 | 0.00 | 0.00 | 0.00 | 0.07 | 0.09 | 0.84 | Bput | ***B. puteoserpentis*** | 0.00 | 1.00 |
| 3676-2 | 3386_B | 0.00 | 0.00 | 0.00 | 0.00 | 0.00 | 0.00 | 0.23 | 0.13 | 0.36 | 0.27 | 0.01 | 0.00 | BC put | **BC *puteoserpentis*** | 0.19 | 0.81 |
| 3676-3 | 3386_C | 0.00 | 0.00 | 0.00 | 0.00 | 0.00 | 0.00 | 1.00 | 0.00 | 0.00 | 0.00 | 0.00 | 0.00 | F2-4 | **F2-4** | 0.32 | 0.68 |
| 3676-4 | 3386_D | 0.00 | 0.00 | 0.00 | 0.00 | 0.00 | 0.00 | 0.00 | 0.00 | 0.00 | 0.04 | 0.07 | 0.89 | Bput | ***B. puteoserpentis*** | 0.00 | 1.00 |
| 3676-5 | 3386_E | 0.00 | 0.00 | 0.00 | 0.00 | 0.00 | 0.00 | 0.00 | 0.00 | 0.00 | 0.07 | 0.08 | 0.84 | Bput | ***B. puteoserpentis*** | 0.00 | 1.00 |
| 3676-6 | 3386_F | 0.00 | 0.00 | 0.00 | 0.00 | 0.00 | 0.00 | 0.00 | 0.00 | 0.02 | 0.28 | 0.16 | 0.54 | BC put | ***B. puteoserpentis*** | 0.01 | 0.99 |
| 3676-7 | 3386_K | 0.00 | 0.00 | 0.00 | 0.00 | 0.00 | 0.00 | 0.00 | 0.00 | 0.13 | 0.50 | 0.17 | 0.20 | BC put | **BC *puteoserpentis*** | 0.04 | 0.96 |
| 3676-8 | 3386_G | 0.00 | 0.01 | 0.00 | 0.00 | 0.00 | 0.60 | 0.36 | 0.04 | 0.00 | 0.00 | 0.00 | 0.00 | F1 | **F2-4** | 0.37 | 0.63 |
| 3676-9 | 3386_H | 0.00 | 0.00 | 0.00 | 0.00 | 0.00 | 0.00 | 1.00 | 0.00 | 0.00 | 0.00 | 0.00 | 0.00 | F2-4 | **F2-4** | 0.28 | 0.72 |
| 3676-10 | 3386_I | 0.00 | 0.00 | 0.00 | 0.00 | 0.00 | 0.00 | 0.00 | 0.00 | 0.00 | 0.07 | 0.09 | 0.84 | Bput | ***B. puteoserpentis*** | 0.00 | 1.00 |
| 3676-11 | 3386_J | 0.00 | 0.00 | 0.00 | 0.00 | 0.00 | 0.00 | 1.00 | 0.00 | 0.00 | 0.00 | 0.00 | 0.00 | F2-4 | **F2-4** | 0.37 | 0.63 |
| 3676-12 | 3386_L | 0.00 | 0.00 | 0.00 | 0.00 | 0.00 | 0.00 | 0.01 | 0.10 | 0.41 | 0.45 | 0.03 | 0.00 | BC put | **BC *puteoserpentis*** | 0.13 | 0.87 |
| 3676-14 | 3386_M | 0.03 | 0.63 | 0.00 | 0.00 | 0.00 | 0.00 | 0.33 | 0.00 | 0.00 | 0.00 | 0.00 | 0.00 | BC azo | **F2-4** | 0.57 | 0.43 |
| 3676-15 | 3386_N | 0.86 | 0.12 | 0.01 | 0.00 | 0.00 | 0.00 | 0.00 | 0.00 | 0.00 | 0.00 | 0.00 | 0.00 | Bazo | **F2-4** | 0.69 | 0.31 |
| 3676-16 | 3386_O | 0.00 | 0.00 | 0.00 | 0.00 | 0.00 | 0.00 | 0.00 | 0.00 | 0.00 | 0.10 | 0.10 | 0.79 | Bput | ***B. puteoserpentis*** | 0.00 | 1.00 |
| 3676-17 | 3386_P | 0.00 | 0.00 | 0.00 | 0.00 | 0.00 | 0.00 | 0.00 | 0.06 | 0.40 | 0.50 | 0.04 | 0.00 | BC put | **BC *puteoserpentis*** | 0.13 | 0.87 |
| 3676-18 | 3386_Q | 0.00 | 0.00 | 0.00 | 0.00 | 0.00 | 0.00 | 0.00 | 0.00 | 0.04 | 0.28 | 0.14 | 0.53 | Bput | **BC *puteoserpentis*** | 0.01 | 0.99 |
| 3676-19 | 3386_R | 0.00 | 0.00 | 0.00 | 0.00 | 0.00 | 0.00 | 0.00 | 0.05 | 0.38 | 0.52 | 0.06 | 0.00 | BC put | **BC *puteoserpentis*** | 0.12 | 0.88 |
| 3676-20 | 3386_S | 0.00 | 0.00 | 0.00 | 0.00 | 0.00 | 0.00 | 0.00 | 0.00 | 0.01 | 0.21 | 0.15 | 0.63 | Bput | ***B. puteoserpentis*** | 0.00 | 1.00 |
| 3676-21 | 3386_T | 0.00 | 0.02 | 0.00 | 0.00 | 0.00 | 0.31 | 0.66 | 0.01 | 0.00 | 0.00 | 0.00 | 0.00 | F2-4 | **F2-4** | 0.40 | 0.60 |
| 3676-22 | 3386_U | 0.00 | 0.00 | 0.00 | 0.00 | 0.00 | 0.20 | 0.59 | 0.19 | 0.03 | 0.00 | 0.00 | 0.00 | F2-4 | **F2-4** | 0.29 | 0.71 |
| 3676-26 | 2339_D | 0.00 | 0.07 | 0.00 | 0.00 | 0.00 | 0.24 | 0.69 | 0.00 | 0.00 | 0.00 | 0.00 | 0.00 | F2-4 | **F2-4** | 0.43 | 0.57 |
| 3676-27 | 3386_V | 0.00 | 0.00 | 0.00 | 0.00 | 0.00 | 0.00 | 0.00 | 0.00 | 0.12 | 0.50 | 0.18 | 0.19 | BC put | **BC *puteoserpentis*** | 0.02 | 0.98 |
| 3676-28 | 2424_E | 0.00 | 0.00 | 0.00 | 0.00 | 0.00 | 0.00 | 1.00 | 0.00 | 0.00 | 0.00 | 0.00 | 0.00 | F2-4 | **F2-4** | 0.36 | 0.64 |
| 3676-29 | 2424_H | 0.00 | 0.00 | 0.00 | 0.00 | 0.00 | 0.00 | 0.00 | 0.00 | 0.02 | 0.19 | 0.13 | 0.66 | Bput | ***B. puteoserpentis*** | 0.00 | 1.00 |
| 3676-30 | 3386_W | 0.01 | 0.56 | 0.00 | 0.00 | 0.00 | 0.00 | 0.43 | 0.00 | 0.00 | 0.00 | 0.00 | 0.00 | BC azo | **F2-4** | 0.54 | 0.46 |
| 3676-31 | 3386_X | 0.15 | 0.79 | 0.01 | 0.00 | 0.00 | 0.00 | 0.05 | 0.00 | 0.00 | 0.00 | 0.00 | 0.00 | BC azo | **F2-4** | 0.63 | 0.37 |
| 3676-32 | 3386_Y | 0.00 | 0.00 | 0.00 | 0.00 | 0.00 | 0.00 | 0.00 | 0.00 | 0.00 | 0.07 | 0.08 | 0.84 | Bput | ***B. puteoserpentis*** | 0.00 | 1.00 |
| 3676-33 | 3386_Z | 0.00 | 0.00 | 0.00 | 0.00 | 0.00 | 0.00 | 0.00 | 0.00 | 0.00 | 0.04 | 0.07 | 0.89 | Bput | ***B. puteoserpentis*** | 0.00 | 1.00 |
| 3676-37 | 3386_AA | 0.00 | 0.00 | 0.00 | 0.00 | 0.00 | 0.00 | 0.02 | 0.17 | 0.43 | 0.36 | 0.01 | 0.00 | BC put | **BC *puteoserpentis*** | 0.17 | 0.83 |
| 3676-38 | 3386_AB | 0.00 | 0.00 | 0.00 | 0.00 | 0.00 | 0.00 | 0.00 | 0.00 | 0.01 | 0.12 | 0.10 | 0.78 | Bput | ***B. puteoserpentis*** | 0.00 | 1.00 |
| 3125-1 | 3386_AC | 0.00 | 0.00 | 0.00 | 0.00 | 0.00 | 0.00 | 0.00 | 0.00 | 0.05 | 0.32 | 0.14 | 0.49 | Bput | ***B. puteoserpentis*** | 0.01 | 0.99 |
| 3125-2 | 3386_AD | 0.02 | 0.82 | 0.00 | 0.00 | 0.00 | 0.01 | 0.15 | 0.00 | 0.00 | 0.00 | 0.00 | 0.00 | BC azo | **F2-4** | 0.57 | 0.43 |
| 3125-3 | 3386_AE | 0.00 | 0.00 | 0.00 | 0.00 | 0.00 | 0.00 | 0.00 | 0.00 | 0.00 | 0.04 | 0.07 | 0.89 | Bput | ***B. puteoserpentis*** | 0.00 | 1.00 |
| 3125-4 | 3386_AF | 0.00 | 0.00 | 0.00 | 0.00 | 0.00 | 0.00 | 0.00 | 0.00 | 0.08 | 0.42 | 0.18 | 0.33 | BC put | **BC *puteoserpentis*** | 0.02 | 0.98 |
| 3125-5 | 3386_AG | 0.00 | 0.04 | 0.00 | 0.00 | 0.00 | 0.06 | 0.90 | 0.00 | 0.00 | 0.00 | 0.00 | 0.00 | F2-4 | **F2-4** | 0.43 | 0.57 |
| 3125-6 | 3386_AH | 0.00 | 0.32 | 0.00 | 0.00 | 0.00 | 0.00 | 0.68 | 0.00 | 0.00 | 0.00 | 0.00 | 0.00 | F2-4 | **F2-4** | 0.51 | 0.49 |
| 3125-7 | 3386_AI | 0.05 | 0.52 | 0.00 | 0.00 | 0.00 | 0.00 | 0.42 | 0.00 | 0.00 | 0.00 | 0.00 | 0.00 | BC azo | **F2-4** | 0.57 | 0.43 |
| 3125-8 | 2424_N | 0.00 | 0.03 | 0.00 | 0.00 | 0.00 | 0.00 | 0.96 | 0.00 | 0.00 | 0.00 | 0.00 | 0.00 | F2-4 | **F2-4** | 0.45 | 0.55 |
| 3125-9 | 3386_AJ | 0.00 | 0.00 | 0.00 | 0.00 | 0.00 | 0.00 | 1.00 | 0.00 | 0.00 | 0.00 | 0.00 | 0.00 | F2-4 | **F2-4** | 0.37 | 0.63 |
| 3125-10 | 3386_AK | 0.00 | 0.00 | 0.00 | 0.00 | 0.00 | 0.00 | 0.00 | 0.00 | 0.01 | 0.11 | 0.10 | 0.78 | Bput | ***B. puteoserpentis*** | 0.00 | 1.00 |
| 3125-11 | 3386_AL | 0.00 | 0.09 | 0.00 | 0.00 | 0.00 | 0.01 | 0.91 | 0.00 | 0.00 | 0.00 | 0.00 | 0.00 | F2-4 | **F2-4** | 0.45 | 0.55 |

**Supplementary Table S 4 | Statistics of SOX symbiont MAGs.** Complete: completeness based on gammaproteobacterial marker genes in CheckM; Contam (Strain): Contamination and percentage of this contamination which can be explained by strain heterogeneity according to CheckM; Contam Corr: Contamination after correction for strain heterogeneity (i.e. strain variants were not considered as contamination).

| **MAG ID** | **Complete [%]** | **Contam (Strain) [%]** | **Contam Corr [%]** | **# Contigs** | **GC [%]** | **Genome size [Mb]** | **Read coverage [x]** |
| --- | --- | --- | --- | --- | --- | --- | --- |
| SOX_BS_2424_D | 94.25 | 0 (0) | 0.00 | 914 | 0.37 | 2.40 | 22 |
| SOX_BS_2424_E | 93.69 | 0.16 (33.33) | 0.11 | 3206 | 0.37 | 2.89 | 100 |
| SOX_BS_2424_H | 92.28 | 0 (0) | 0.00 | 772 | 0.37 | 2.13 | 17 |
| SOX_BS_2424_N | 92.28 | 6.87 (95.45) | 0.31 | 4717 | 0.36 | 3.57 | 1001 |
| SOX_BS_3386_A | 94.44 | 1.11 (66.67) | 0.37 | 3998 | 0.37 | 2.76 | 241 |
| SOX_BS_3386_AB | 94.25 | 1.64 (100) | 0.00 | 4095 | 0.37 | 2.80 | 372 |
| SOX_BS_3386_AD | 94.25 | 0.14 (100) | 0.00 | 3928 | 0.37 | 2.69 | 472 |
| SOX_BS_3386_AE | 94.25 | 0.28 (100) | 0.00 | 3899 | 0.37 | 2.73 | 105 |
| SOX_BS_3386_AG | 95.09 | 7.24 (84) | 1.16 | 4767 | 0.36 | 3.46 | 1744 |
| SOX_BS_3386_AH | 94.25 | 3.5 (12.5) | 3.06 | 4113 | 0.36 | 3.44 | 542 |
| SOX_BS_3386_AJ | 92 | 8.81 (86.11) | 1.22 | 5570 | 0.36 | 4.04 | 989 |
| SOX_BS_3386_AK | 94.63 | 0.45 (40) | 0.27 | 3648 | 0.37 | 2.70 | 301 |
| SOX_BS_3386_AL | 94.25 | 3.04 (58.33) | 1.27 | 3415 | 0.37 | 2.62 | 387 |
| SOX_BS_3386_C | 94.25 | 3.51 (8.33) | 3.22 | 3038 | 0.37 | 2.50 | 502 |
| SOX_BS_3386_D | 94.25 | 2.09 (25) | 1.57 | 4408 | 0.37 | 2.92 | 432 |
| SOX_BS_3386_E | 90.18 | 0 (0) | 0.00 | 143 | 0.38 | 1.43 | 488 |
| SOX_BS_3386_F | 94.25 | 2.2 (100) | 0.00 | 4006 | 0.37 | 2.70 | 382 |
| SOX_BS_3386_G | 94.25 | 2.57 (90.91) | 0.23 | 3656 | 0.37 | 2.63 | 601 |
| SOX_BS_3386_H | 89.05 | 0 (0) | 0.00 | 145 | 0.38 | 1.37 | 527 |
| SOX_BS_3386_I | 92.43 | 0 (0) | 0.00 | 157 | 0.38 | 1.48 | 365 |
| SOX_BS_3386_J | 91.3 | 0 (0) | 0.00 | 163 | 0.38 | 1.48 | 2588 |
| SOX_BS_3386_M | 94.25 | 4.45 (33.33) | 2.97 | 3425 | 0.37 | 2.70 | 2704 |
| SOX_BS_3386_O | 94.81 | 3.79 (36.36) | 2.41 | 4500 | 0.37 | 3.07 | 515 |
| SOX_BS_3386_S | 94.25 | 4.03 (69.23) | 1.24 | 3607 | 0.37 | 2.90 | 489 |
| SOX_BS_3386_T | 93.97 | 4.35 (63.64) | 1.58 | 2934 | 0.37 | 2.60 | 298 |
| SOX_BS_3386_U | 94.25 | 0.14 (100) | 0.00 | 4081 | 0.37 | 2.81 | 249 |
| SOX_BS_3386_W | 94.25 | 2.2 (87.5) | 0.28 | 3531 | 0.37 | 2.72 | 398 |
| SOX_BS_3386_X | 94.25 | 4.57 (28.57) | 3.21 | 3161 | 0.37 | 2.79 | 419 |
| SOX_BS_3386_Y | 94.25 | 0.14 (100) | 0.00 | 3032 | 0.37 | 2.66 | 431 |
| SOX_BS_3386_Z | 94.53 | 3.31 (61.54) | 1.27 | 4674 | 0.36 | 3.48 | 951 |

**Supplementary Table S 5 | List of external data.** BioProject and dataset accessions for the European Nucleotide Archive and the respective publication were listed if available. Lat: Latitude; Lon: Longitude; Complete: completeness based on gammaproteobacterial marker genes in CheckM; Contam: Contamination; Strain: Percentage of this contamination which can be explained by strain heterogeneity according to CheckM; Pub: Publication.

| **MAG** | **Site** | **Lat** | **Lon** | **Cruise** | **Host species** | **Complete** | **Contam** | **Strain** | **Pub** | **BioProject** | **Accession** |
| --- | --- | --- | --- | --- | --- | --- | --- | --- | --- | --- | --- |
| 1048F | Lucky Strike (Montsegur) | 37.288 | -32.276 | Biobaz (2013) | *B. azoricus* | 94.53 | 0 | 0 | [92] | PRJEB36091 | GCA_903813355 |
| 1048G | Lucky Strike (Montsegur) | 37.288 | -32.276 | Biobaz (2013) | *B. azoricus* | 94.53 | 0 | 0 | [92] | PRJEB36091 | GCA_903813365 |
| 1048H | Lucky Strike (Montsegur) | 37.288 | -32.276 | Biobaz (2013) | *B. azoricus* | 95.09 | 0 | 0 | [92] | PRJEB36091 | GCA_903819415 |
| 1048I | Lucky Strike (Eiffel Tower) | 37.283 | -32.276 | Biobaz (2013) | *B. azoricus* | 93.97 | 0.03 | 0 | [92] | PRJEB36091 | GCA_903819345 |
| 1048J | Lucky Strike (Eiffel Tower) | 38.283 | -32.276 | Biobaz (2013) | *B. azoricus* | 94.53 | 0.56 | 100 | [92] | PRJEB36091 | GCA_903819365 |
| 1586B | Lucky Strike (Eiffel Tower) | 37.289 | -32.275 | Biobaz (2013) | *B. azoricus* | 93.41 | 0.84 | 100 | [92] | PRJEB36091 | GCA_903813405 |
| 1586C | Lucky Strike (Eiffel Tower) | 37.289 | -32.275 | Biobaz (2013) | *B. azoricus* | 92.85 | 0.84 | 50 | [92] | PRJEB36091 | GCA_903819425 |
| 1586D | Lucky Strike (Eiffel Tower) | 37.289 | -32.275 | Biobaz (2013) | *B. azoricus* | 93.97 | 0 | 0 | [92] | PRJEB36091 | GCA_903813415 |
| 1586E | Lucky Strike (Eiffel Tower) | 37.289 | -32.275 | Biobaz (2013) | *B. azoricus* | 92.85 | 0 | 0 | [92] | PRJEB36091 | GCA_903813425 |
| 1586F | Lucky Strike (Eiffel Tower) | 37.283 | -32.276 | Biobaz (2013) | *B. azoricus* | 92.85 | 1.4 | 33.3 | [92] | PRJEB36091 | GCA_903813665 |
| 1586G | Lucky Strike (Eiffel Tower) | 37.283 | -32.276 | Biobaz (2013) | *B. azoricus* | 93.97 | 0 | 0 | [92] | PRJEB36091 | GCA_903813675 |
| 1586I | Lucky Strike (Eiffel Tower) | 37.283 | -32.276 | Biobaz (2013) | *B. azoricus* | 94.53 | 0 | 0 | [93] |  | SAMEA6822959 |
| 1586J | Lucky Strike (Eiffel Tower) | 37.283 | -32.276 | Biobaz (2013) | *B. azoricus* | 93.97 | 0.56 | 0 | [92] | PRJEB36091 | GCA_903799955 |
| 1586K | Lucky Strike (Montsegur) | 37.288 | -32.276 | Biobaz (2013) | *B. azoricus* | 92.28 | 0.56 | 0 | [92] | PRJEB36091 | GCA_903813645 |
| 1586N | Lucky Strike (Montsegur) | 37.288 | -32.276 | Biobaz (2013) | *B. azoricus* | 94.53 | 0 | 0 | [92] | PRJEB36091 | GCA_903813615 |
| 1586O | Lucky Strike (Montsegur) | 37.288 | -32.276 | Biobaz (2013) | *B. azoricus* | 93.6 | 1.12 | 50 | [92] | PRJEB36091 | GCA_903813655 |
| 1586P | Menez Gwen (White Flames) | 37.844 | -31.519 | Biobaz (2013) | *B. azoricus* | 91.72 | 0 | 0 | [92] | PRJEB36091 | GCA_903813625 |
| 1586Q | Menez Gwen (White Flames) | 37.844 | -31.519 | Biobaz (2013) | *B. azoricus* | 92.28 | 0 | 0 | [92] | PRJEB36091 | GCA_903813695 |
| 1586R | Menez Gwen (White Flames) | 37.844 | -31.519 | Biobaz (2013) | *B. azoricus* | 93.97 | 0 | 0 | [93] |  | SAMEA6822960 |
| 1586S | Menez Gwen (White Flames) | 37.844 | -31.519 | Biobaz (2013) | *B. azoricus* | 93.97 | 0 | 0 | [92] | PRJEB36091 | GCA_903813685 |
| 1600F | Rainbow | 36.229 | -33.902 | Biobaz (2013) | *B. azoricus* | 93.97 | 0 | 0 | [92] | PRJEB36091 | GCA_903813635 |
| 1600G | Rainbow | 36.229 | -33.902 | Biobaz (2013) | *B. azoricus* | 93.97 | 0 | 0 | [92] | PRJEB36091 | GCA_903813705 |
| 1600H | Rainbow | 36.229 | -33.902 | Biobaz (2013) | *B. azoricus* | 93.93 | 0 | 0 | [92] | PRJEB36091 | GCA_903819385 |
| 1600I | Rainbow | 36.229 | -33.902 | Biobaz (2013) | *B. azoricus* | 91.72 | 0 | 0 | [92] | PRJEB36091 | GCA_903813715 |
| 1600J | Rainbow | 36.229 | -33.902 | Biobaz (2013) | *B. azoricus* | 93.97 | 0.14 | 100 | [92] | PRJEB36091 | GCA_903813725 |
| BBROOKSOX | Chapopote (Mexico) | 21.90002 | -93.4353 | M114-2 | *B. brooksi* | 93.27 | 0.56 | 0 | [93] | PRJEB17996 | GCA_900128405.1 |
| BHECKSOX | Chapopote (Mexico) | 21.90005 | -93.4354 | M114-2 | *B. heckerae* | 92.66 | 4.31 | 63.6 | [93] | PRJEB17996 | GCA_900128515.1 |
| 1115A | Semenov | 13.513 | -44.963 | Odemar (2014) | *B. puteoserpentis* | 94.53 | 3.09 | 75 | [92] | PRJEB36091 | GCA_903813375 |
| 1115B | Semenov | 13.513 | -44.963 | Odemar (2014) | *B. puteoserpentis* | 93.41 | 3.09 | 85.7 | [92] | PRJEB36091 | GCA_903813385 |
| 1115C | Semenov | 13.513 | -44.963 | Odemar (2014) | *B. puteoserpentis* | 94.53 | 0.56 | 100 | [92] | PRJEB36091 | GCA_903813395 |
| 2065A | Logatchev Quest | 14.753 | -44.979 | M64-2 Logatchev (2005) | *B. puteoserpentis* | 94.72 | 7.3 | 100 | [92] | PRJEB36091 | GCA_903813905 |
| 2065B | Logatchev Quest | 14.753 | -44.979 | M64-2 Logatchev (2005) | *B. puteoserpentis* | 94.72 | 1.15 | 100 | [92] | PRJEB36091 | GCA_903813925 |
| 2487A | Logatchev Quest | 14.753 | -44.980 | M126 (2016) | *B. puteoserpentis* | 94.16 | 1.4 | 57.1 | [92] | PRJEB36091 | GCA_903819405 |
| 2487B | Logatchev Quest | 14.753 | -44.980 | M126 (2016) | *B. puteoserpentis* | 94.72 | 1.4 | 57.1 | [92] | PRJEB36091 | GCA_903819245 |
| 2487C | Logatchev Quest | 14.753 | -44.980 | M126 (2016) | *B. puteoserpentis* | 94.72 | 1.4 | 83.3 | [92] | PRJEB36091 | GCA_903813875 |
| 2487D | Semenov | 13.514 | -44.963 | M126 (2016) | *B. puteoserpentis* | 94.53 | 1.12 | 66.7 | [92] | PRJEB36091 | GCA_903813855 |
| 2487E | Semenov | 13.514 | -44.963 | M126 (2016) | *B. puteoserpentis* | 94.72 | 1.12 | 66.7 | [92] | PRJEB36091 | GCA_903819375 |
| 2487F | Semenov | 13.514 | -44.963 | M126 (2016) | *B. puteoserpentis* | 94.53 | 1.97 | 83.3 | [92] | PRJEB36091 | GCA_903813935 |
| 3722CJ | Logatchev Quest | 14.753 | -44.981 | MSM10-03 Hydromar VII | *B. puteoserpentis* | 94.72 | 5.15 | 50 | [92] | PRJEB36091 | GCA_903814125 |
| 3722CK | Logatchev Quest | 14.753 | -44.981 | MSM10-03 Hydromar VII | *B. puteoserpentis* | 94.72 | 3.65 | 78.6 | [92] | PRJEB36091 | GCA_903819395 |
| 3722CL | Logatchev Quest | 14.753 | -44.981 | MSM10-03 Hydromar VII | *B. puteoserpentis* | 94.72 | 3.93 | 92.9 | [92] | PRJEB36091 | GCA_903814105 |
| 3722CM | Logatchev Quest | 14.753 | -44.981 | MSM10-03 Hydromar VII | *B. puteoserpentis* | 94.72 | 3.23 | 100 | [92] | PRJEB36091 | GCA_903814155 |
| 3722CN | Logatchev Quest | 14.753 | -44.981 | MSM10-03 Hydromar VII | *B. puteoserpentis* | 93.6 | 2.39 | 76.9 | [92] | PRJEB36091 | GCA_903814145 |
| 3722CO | Logatchev Quest | 14.753 | -44.981 | MSM10-03 Hydromar VII | *B. puteoserpentis* | 93.03 | 2.81 | 92.3 | [92] | PRJEB36091 | GCA_903814115 |
| 3722CP | Logatchev Quest | 14.753 | -44.980 | M126 (2016) | *B. puteoserpentis* | 94.72 | 8.8 | 90.9 | [92] | PRJEB36091 | GCA_903814135 |
| Endosymbiont of *Bathymodiolus septemdierum* str. Myojin knoll DNA, complete genome | Izu-Bonin Arc, Myojin knoll (Japan) | 32.104 | 139.219 |  | *B. septemdierum* | 94.83 | 0.56 | 100 | [56] | PRJDB949 | NZ_AP013042.1 |
| C112 | Clueless | -4.803 | -12.372 | M78-2 (2009) | *B.* sp. Clueless | 94.53 | 1.31 | 75 | [92] | PRJEB36091 | GCA_903814195 |
| C113 | Clueless | -4.803 | -12.372 | M78-2 (2009) | *B.* sp. Clueless | 94.53 | 1.4 | 66.7 | [92] | PRJEB36091 | GCA_903814185 |
| C114 | Clueless | -4.803 | -12.372 | M78-2 (2009) | *B.* sp. Clueless | 94.53 | 1.4 | 66.7 | [92] | PRJEB36091 | GCA_903814175 |
| L102 | Lilliput | -9.547 | -13.210 | M78-2 (2009) | *B.* sp. Lilliput | 93.33 | 1.12 | 100 | [92] | PRJEB36091 | GCA_903813445 |
| L51 | Lilliput | -9.547 | -13.210 | M78-2 (2009) | *B.* sp. Lilliput | 94.08 | 1.69 | 100 | [92] | PRJEB36091 | GCA_903813485 |
| L54 | Lilliput | -9.547 | -13.210 | M78-2 (2009) | *B.* sp. Lilliput | 94.36 | 0.84 | 100 | [92] | PRJEB36091 | GCA_903813455 |
| *Bathymodiolus thermophilus* thioautotrophic gill symbiont strain:BAT/CrabSpa'14 | East Pacific Rise (EPR) 9°N | 9.839833 | -104.292 | R/V *Atlantis* cruise AT26–10 | *B. thermophilus* | 96.98 | 11.32 | 81.4 | [94] | PRJNA339702 | GCA_001875585 |
| *Ca.* Ruthia magnifica str. Cm | 9° East Pacific Rise vent field | 9.830 | -104.290 |  | *C. magnifica* | 86.67 | 0 | 0 | [95] | PRJNA16841 | CP000488 |
| *Ca.* Vesicomyosocius okutanii HA | Sagami Bay | 35.117 | 139.383 |  | *C. okutanii* | 85.69 | 0 | 0 | [96] | PRJDA18267 | AP009247 |
| *Ca.* Thioglobus autotrophicus strain EF1 | Effingham Inlet (estimated coordinates) | 49.029 | -125.154 |  |  | 94.64 | 0 | 0 | [97] | PRJNA224116 | NZ_CP010552 |
| *Ca.* Thioglobus singularis PS1 | Puget Sound | 47.600 | -122.450 |  |  | 94.36 | 0 | 0 | [98] | PRJNA229178 | CP006911 |
| *Thiomicrospira crunogena* XCL-2 (*Hydrogenovibrio crunogenus* XCL-2) |  |  |  |  |  | 99.72 | 0.19 | 0 | [99] | PRJNA13018 | NC_007520.2 |

# References

1. Won Y-J, Hallam SJ, O’Mullan GD, Pan IL, Buck KR, Vrijenhoek RC. Environmental acquisition of thiotrophic endosymbionts by deep-sea mussels of the genus *Bathymodiolus*. Appl Environ Microbiol. 2003; 69: 6785–92.

2. RStudio Team. RStudio: integrated development for R. Boston, MA: RStudio, Inc.; 2015. http://www.rstudio.com/ (accessed December 19, 2016).

3. South A. rnaturalearth: World map data from Natural Earth. 2017. https://CRAN.R-project.org/package=rnaturalearth (accessed June 23, 2020).

4. Gallic E. legendMap: north arrow and scale bar for ggplot2 graphics. 2016. https://rdrr.io/github/3wen/legendMap/man/legendMap-package.html (accessed June 23, 2020).

5. Wickham H, Chang W, Henry L, Pedersen TL, Takahashi K, Wilke C, et al. ggplot2: create elegant data visualisations using the grammar of graphics. 2019. https://CRAN.R-project.org/package=ggplot2 (accessed November 25, 2019).

6. Falush D, Stephens M, Pritchard JK. Inference of population structure using multilocus genotype data: linked loci and correlated allele frequencies. Genetics. 2003; 164: 1567–87.

7. Pritchard JK, Stephens M, Donnelly P. Inference of population structure using multilocus genotype data. Genetics. 2000; 155: 945–59.

8. Hubisz MJ, Falush D, Stephens M, Pritchard JK. Inferring weak population structure with the assistance of sample group information. Mol Ecol Resour. 2009; 9: 1322–32.

9. Chhatre VE, Emerson KJ. StrAuto: Automation and parallelization of STRUCTURE analysis. BMC Bioinformatics. 2017; 18: 192.

10. CLUMPAK server. n.d. http://clumpak.tau.ac.il/ (accessed November 25, 2019).

11. Anderson EC, Thompson EA. A model-based method for identifying species hybrids using multilocus genetic data. Genetics. 2002; 160: 1217–29.

12. Gompert Z, Buerkle CA. introgress: a software package for mapping components of isolation in hybrids. Mol Ecol Resour. 2010; 10: 378–84.

13. Gompert Z, Buerkle CA. A powerful regression-based method for admixture mapping of isolation across the genome of hybrids. Mol Ecol. 2009; 18: 1207–24.

14. Breusing C, Biastoch A, Drews A, Metaxas A, Jollivet D, Vrijenhoek RC, et al. Biophysical and population genetic models predict the presence of “phantom” stepping stones connecting Mid-Atlantic Ridge vent ecosystems. Curr Biol. 2016; 26: 2257–67.

15. Breusing C, Vrijenhoek RC, Reusch TBH. Widespread introgression in deep-sea hydrothermal vent mussels. BMC Evol Biol. 2017; 17: 13.

16. NCBI Resource Coordinators. Database resources of the National Center for Biotechnology Information. Nucleic Acids Res. 2016; 44: D7–19.

17. Edgar RC. MUSCLE: a multiple sequence alignment method with reduced time and space complexity. BMC Bioinformatics. 2004; 5: 113.

18. Edgar RC. MUSCLE: multiple sequence alignment with high accuracy and high throughput. Nucleic Acids Res. 2004; 32: 1792–7.

19. Nguyen L-T, Schmidt HA, von Haeseler A, Minh BQ. IQ-TREE: a fast and effective stochastic algorithm for estimating maximum-likelihood phylogenies. Mol Biol Evol. 2015; 32: 268–74.

20. Kalyaanamoorthy S, Minh BQ, Wong TKF, von Haeseler A, Jermiin LS. ModelFinder: fast model selection for accurate phylogenetic estimates. Nat Methods. 2017; 14: 587–9.

21. Minh BQ, Nguyen MAT, von Haeseler A. Ultrafast approximation for phylogenetic bootstrap. Mol Biol Evol. 2013; 30: 1188–95.

22. Rota-Stabelli O, Yang Z, Telford MJ. MtZoa: a general mitochondrial amino acid substitutions model for animal evolutionary studies. Mol Phylogenet Evol. 2009; 52: 268–72.

23. Letunic I, Bork P. Interactive Tree Of Life (iTOL) v4: recent updates and new developments. Nucleic Acids Res. 2019; 47: W256–9.

24. Adobe. Adobe Illustrator. 2020. https://www.adobe.com/de/products/illustrator.html (accessed February 3, 2020).

25. Bushnell B. BBMap. 2014. http://sourceforge.net/projects/bbmap/ (accessed October 21, 2016).

26. Li D, Liu C-M, Luo R, Sadakane K, Lam T-W. MEGAHIT: an ultra-fast single-node solution for large and complex metagenomics assembly via succinct de Bruijn graph. Bioinformatics. 2015; 31: 1674–6.

27. Li D, Luo R, Liu C-M, Leung C-M, Ting H-F, Sadakane K, et al. MEGAHIT v1.0: a fast and scalable metagenome assembler driven by advanced methodologies and community practices. Methods. 2016; 102: 3–11.

28. Kang DD, Froula J, Egan R, Wang Z. MetaBAT, an efficient tool for accurately reconstructing single genomes from complex microbial communities. PeerJ. 2015; 3: e1165.

29. Li H, Handsaker B, Wysoker A, Fennell T, Ruan J, Homer N, et al. The sequence alignment/map format and SAMtools. Bioinformatics. 2009; 25: 2078–9.

30. Seemann T. barrnap. 2014. http://www.vicbioinformatics.com/software.barrnap.shtml (accessed October 21, 2016).

31. Rognes T, Flouri T, Nichols B, Quince C, Mahé F. VSEARCH: a versatile open source tool for metagenomics. PeerJ. 2016; 4: e2584.

32. Edgar RC. Usearch. 2010. http://www.drive5.com/usearch/ (accessed October 21, 2016).

33. Quast C, Pruesse E, Yilmaz P, Gerken J, Schweer T, Yarza P, et al. The SILVA ribosomal RNA gene database project: improved data processing and web-based tools. Nucleic Acids Res. 2013; 41: D590–6.

34. Wu M, Scott AJ. Phylogenomic analysis of bacterial and archaeal sequences with AMPHORA2. Bioinformatics. 2012; 28: 1033–4.

35. Seah BKB, Gruber-Vodicka HR. gbtools: Interactive visualization of metagenome bins in R. Front Microbiol. 2015; 6: 1451.

36. Parks DH, Imelfort M, Skennerton CT, Hugenholtz P, Tyson GW. CheckM: assessing the quality of microbial genomes recovered from isolates, single cells, and metagenomes. Genome Res. 2015; 25: 1043–55.

37. Matsen FA, Kodner RB, Armbrust EV. pplacer: linear time maximum-likelihood and Bayesian phylogenetic placement of sequences onto a fixed reference tree. BMC Bioinformatics. 2010; 11: 538.

38. Hyatt D, Chen G-L, LoCascio PF, Land ML, Larimer FW, Hauser LJ. Prodigal: prokaryotic gene recognition and translation initiation site identification. BMC Bioinformatics. 2010; 11: 119.

39. HMMER. n.d. http://hmmer.org/ (accessed November 25, 2019).

40. Ansorge R, Romano S, Sayavedra L, González Porras MÁ, Kupczok A, Tegetmeyer HE, et al. Functional diversity enables multiple symbiont strains to coexist in deep-sea mussels. Nat Microbiol. 2019; 4: 2487–97.

41. Bankevich A, Nurk S, Antipov D, Gurevich AA, Dvorkin M, Kulikov AS, et al. SPAdes: a new genome assembly algorithm and its applications to single-cell sequencing. J Comput Biol. 2012; 19: 455–77.

42. Wick RR, Schultz MB, Zobel J, Holt KE. Bandage: interactive visualization of de novo genome assemblies. Bioinformatics. 2015; 31: 3350–2.

43. Lee MD. GToTree: a user-friendly workflow for phylogenomics. Bioinformatics. 2019; 35: 4162–4.

44. Capella-Gutiérrez S, Silla-Martínez JM, Gabaldón T. trimAl: a tool for automated alignment trimming in large-scale phylogenetic analyses. Bioinformatics. 2009; 25: 1972–3.

45. Kearse M, Moir R, Wilson A, Stones-Havas S, Cheung M, Sturrock S, et al. Geneious Basic: an integrated and extendable desktop software platform for the organization and analysis of sequence data. Bioinformatics. 2012; 28: 1647–9.

46. Le SQ, Gascuel O. An improved general amino acid replacement matrix. Mol Biol Evol. 2008; 25: 1307–20.

47. Jombart T, Kamvar ZN, Collins C, Lustrik R, Beugin M-P, Knaus BJ, et al. adegenet: exploratory analysis of genetic and genomic data. 2020. https://CRAN.R-project.org/package=adegenet (accessed February 6, 2020).

48. Charif D, Clerc O, Frank C, Lobry JR, Necşulea A, Palmeira L, et al. seqinr: biological sequences retrieval and analysis. 2019. https://CRAN.R-project.org/package=seqinr (accessed February 20, 2020).

49. Goudet J, Jombart T. hierfstat: estimation and tests of hierarchical F-statistics. 2015. https://CRAN.R-project.org/package=hierfstat (accessed February 20, 2020).

50. Meirmans PG. The trouble with isolation by distance. Mol Ecol. 2012; 21: 2839–46.

51. Oksanen J, Blanchet FG, Friendly M, Kindt R, Legendre P, McGlinn D, et al. vegan: Community ecology package. 2019. https://CRAN.R-project.org/package=vegan (accessed November 25, 2019).

52. Jain C, Rodriguez-R LM, Phillippy AM, Konstantinidis KT, Aluru S. High throughput ANI analysis of 90K prokaryotic genomes reveals clear species boundaries. Nat Commun. 2018; 9: 5114.

53. Warnes GR, Bolker B, Bonebakker L, Gentleman R, Liaw WHA, Lumley T, et al. gplots: various R programming tools for plotting data. 2019. https://CRAN.R-project.org/package=gplots (accessed November 25, 2019).

54. Demin G. maditr: fast data aggregation, modification, and filtering with pipes and “data.table.” 2019. https://CRAN.R-project.org/package=maditr (accessed November 25, 2019).

55. Paradis E, Blomberg S, Bolker B, Brown J, Claude J, Cuong HS, et al. ape: Analyses of phylogenetics and evolution. 2019. https://CRAN.R-project.org/package=ape (accessed February 6, 2020).

56. Ikuta T, Takaki Y, Nagai Y, Shimamura S, Tsuda M, Kawagucci S, et al. Heterogeneous composition of key metabolic gene clusters in a vent mussel symbiont population. ISME J. 2016; 10: 990–1001.

57. Picazo DR, Dagan T, Ansorge R, Petersen JM, Dubilier N, Kupczok A. Horizontally transmitted symbiont populations in deep-sea mussels are genetically isolated. ISME J. 2019; 13: 2954–2968.

58. Seemann T. Prokka: rapid prokaryotic genome annotation. Bioinformatics. 2014; 30: 2068–9.

59. Broad Institute. Picard Tools. 2013. https://github.com/broadinstitute/picard (accessed January 17, 2017).

60. McKenna A, Hanna M, Banks E, Sivachenko A, Cibulskis K, Kernytsky A, et al. The Genome Analysis Toolkit: a MapReduce framework for analyzing next-generation DNA sequencing data. Genome Res. 2010; 20: 1297–303. http://www.ncbi.nlm.nih.gov/pmc/articles/PMC2928508/ (accessed January 17, 2017).

61. Contreras-Moreira B, Vinuesa P. GET_HOMOLOGUES, a versatile software package for scalable and robust microbial pangenome analysis. Appl Environ Microbiol. 2013; 79: 7696–701.

62. Vinuesa P, Contreras-Moreira B. Robust identification of orthologues and paralogues for microbial pan-genomics using GET_HOMOLOGUES: a case study of pIncA/C plasmids. In: Mengoni A, Galardini M, Fondi M, editors. Bact. Pangenomics, vol. 1231, New York, NY: Springer; 2015, p. 203–32.

63. Li L, Stoeckert CJ, Roos DS. OrthoMCL: Identification of ortholog groups for eukaryotic genomes. Genome Res. 2003; 13: 2178–89.

64. Kristensen DM, Kannan L, Coleman MK, Wolf YI, Sorokin A, Koonin EV, et al. A low-polynomial algorithm for assembling clusters of orthologous groups from intergenomic symmetric best matches. Bioinformatics. 2010; 26: 1481–7.

65. Altschul SF, Madden TL, Schäffer AA, Zhang J, Zhang Z, Miller W, et al. Gapped BLAST and PSI-BLAST: a new generation of protein database search programs. Nucleic Acids Res. 1997; 25: 3389–402.

66. Stajich JE, Block D, Boulez K, Brenner SE, Chervitz SA, Dagdigian C, et al. The Bioperl Toolkit: Perl modules for the life sciences. Genome Res. 2002; 12: 1611–8.

67. Buchfink B, Xie C, Huson DH. Fast and sensitive protein alignment using DIAMOND. Nat Methods. 2015; 12: 59–60.

68. Finn RD, Coggill P, Eberhardt RY, Eddy SR, Mistry J, Mitchell AL, et al. The Pfam protein families database: towards a more sustainable future. Nucleic Acids Res. 2016; 44: D279–85.

69. Brown NP, Leroy C, Sander C. MView: a web-compatible database search or multiple alignment viewer. Bioinformatics. 1998; 14: 380–1.

70. Bray NL, Pimentel H, Melsted P, Pachter L. Near-optimal probabilistic RNA-seq quantification. Nat Biotechnol. 2016; 34: 525–7.

71. Grabherr MG, Haas BJ, Yassour M, Levin JZ, Thompson DA, Amit I, et al. Trinity: reconstructing a full-length transcriptome without a genome from RNA-Seq data. Nat Biotechnol. 2011; 29: 644–52.

72. Haas BJ, Papanicolaou A, Yassour M, Grabherr M, Blood PD, Bowden J, et al. De novo transcript sequence reconstruction from RNA-seq using the Trinity platform for reference generation and analysis. Nat Protoc. 2013; 8: 1494–512.

73. Fernandes AD, Macklaim JM, Linn TG, Reid G, Gloor GB. ANOVA-like differential expression (ALDEx) analysis for mixed population RNA-Seq. PLOS ONE. 2013; 8: e67019.

74. Fernandes AD, Reid JN, Macklaim JM, McMurrough TA, Edgell DR, Gloor GB. Unifying the analysis of high-throughput sequencing datasets: characterizing RNA-Seq, 16S rRNA gene sequencing and selective growth experiments by compositional data analysis. Microbiome. 2014; 2: 15.

75. Gloor GB, Macklaim JM, Fernandes AD. Displaying variation in large datasets: plotting a visual summary of effect sizes. J Comput Graph Stat. 2016; 25: 971–9.

76. Dowle M, Srinivasan A, Gorecki J, Chirico M, Stetsenko P, Short T, et al. data.table: extension of “data.frame.” 2019. https://CRAN.R-project.org/package=data.table (accessed November 25, 2019).

77. Borcard D, Gillet F, Legendre P. Numerical ecology with R. Springer; 2018.

78. Amend JP, McCollom TM, Hentscher M, Bach W. Catabolic and anabolic energy for chemolithoautotrophs in deep-sea hydrothermal systems hosted in different rock types. Geochim Cosmochim Acta. 2011; 75: 5736–48.

79. Ter Braak CJF. The analysis of vegetation-environment relationships by canonical correspondence analysis. Vegetatio. 1987; 69: 69–77.

80. Legendre P. Spatial autocorrelation: Trouble or new paradigm? Ecology. 1993; 74: 1659–73.

81. Beck MW. ggord: ordination plots with ggplot2. 2019. https://rdrr.io/github/fawda123/ggord/man/ggord.html (accessed June 23, 2020).

82. Arnold ML, Hodges SA. Are natural hybrids fit or unfit relative to their parents? Trends Ecol Evol. 1995; 10: 67–71.

83. Colaço A, Martins I, Laranjo M, Pires L, Leal C, Prieto C, et al. Annual spawning of the hydrothermal vent mussel, *Bathymodiolus azoricus*, under controlled aquarium conditions at atmospheric pressure. J Exp Mar Biol Ecol. 2006; 333: 166–71.

84. Miyake H, Kitada M, Itoh T, Nemoto S, Okuyama Y, Watanabe H, et al. Larvae of deep-sea chemosynthetic ecosystem animals in captivity. Cah Biol Mar. 2010; 51: 441–50.

85. Johnson SB, Won Y-J, Harvey JB, Vrijenhoek RC. A hybrid zone between *Bathymodiolus* mussel lineages from eastern Pacific hydrothermal vents. BMC Evol Biol. 2013; 13: 21.

86. Bordenstein SR, O’Hara FP, Werren JH. *Wolbachia*-induced incompatibility precedes other hybrid incompatibilities in *Nasonia*. Nature. 2001; 409: 707–10.

87. Breeuwer JAJ, Werren JH. Hybrid breakdown between two haplodiploid species: the role of nuclear and cytoplasmic genes. Evolution. 1995; 49: 705–17.

88. Coyne JA. Genetics and speciation. Nature. 1992; 355: 511–5.

89. Wu CI, Davis AW. Evolution of postmating reproductive isolation: the composite nature of Haldane’s rule and its genetic bases. Am Nat. 1993; 142: 187–212.

90. Wright S. Isolation by distance. Genetics. 1943; 28: 114–38. https://www.ncbi.nlm.nih.gov/pmc/articles/PMC1209196/ (accessed June 19, 2020).

91. Meirmans PG. Seven common mistakes in population genetics and how to avoid them. Mol Ecol. 2015; 24: 3223–31.

92. Ansorge R, Romano S, Sayavedra L, Rubin-Blum M, Gruber-Vodicka H, Scilipoti S, et al. The hidden pangenome: comparative genomics reveals pervasive diversity in symbiotic and free-living sulfur-oxidizing bacteria. BioRxiv. 2020: 2020.12.11.421487.

93. Sayavedra L, Ansorge R, Rubin-Blum M, Leisch N, Dubilier N, Petersen JM. Horizontal acquisition followed by expansion and diversification of toxin-related genes in deep-sea bivalve symbionts. BioRxiv. 2019: 605386.

94. Ponnudurai R, Sayavedra L, Kleiner M, Heiden SE, Thürmer A, Felbeck H, et al. Genome sequence of the sulfur-oxidizing *Bathymodiolus thermophilus* gill endosymbiont. Stand Genomic Sci. 2017; 12: 50.

95. Newton ILG, Woyke T, Auchtung TA, Dilly GF, Dutton RJ, Fisher MC, et al. The *Calyptogena magnifica* chemoautotrophic symbiont genome. Science. 2007; 315: 998–1000.

96. Kuwahara H, Yoshida T, Takaki Y, Shimamura S, Nishi S, Harada M, et al. Reduced genome of the thioautotrophic intracellular symbiont in a deep-sea clam, *Calyptogena okutanii*. Curr Biol. 2007; 17: 881–6.

97. Shah V, Morris RM. Genome sequence of “*Candidatus* Thioglobus autotrophica” strain EF1, a chemoautotroph from the SUP05 clade of marine Gammaproteobacteria. Genome Announc. 2015; 3: e01156-15.

98. Marshall KT, Morris RM. Genome sequence of “*Candidatus* Thioglobus singularis” strain PS1, a mixotroph from the SUP05 clade of marine Gammaproteobacteria. Genome Announc. 2015; 3: e01155-15.

99. Scott KM, Sievert SM, Abril FN, Ball LA, Barrett CJ, Blake RA, et al. The genome of deep-sea vent chemolithoautotroph *Thiomicrospira crunogena* XCL-2. PLoS Biol. 2006; 4: e383.
